# Neighborhood Resources Associated With Psychological Trajectories and Neural Reactivity to Reward After Trauma

**DOI:** 10.1001/jamapsychiatry.2024.2148

**Published:** 2024-07-31

**Authors:** E. Kate Webb, Jennifer S. Stevens, Timothy D. Ely, Lauren A. M. Lebois, Sanne J H. van Rooij, Steven E. Bruce, Stacey L. House, Francesca L. Beaudoin, Xinming An, Thomas C. Neylan, Gari D. Clifford, Sarah D. Linnstaedt, Laura T. Germine, Kenneth A. Bollen, Scott L. Rauch, John P. Haran, Alan B. Storrow, Christopher Lewandowski, Paul I. Musey, Phyllis L. Hendry, Sophia Sheikh, Christopher W. Jones, Brittany E. Punches, Robert A. Swor, Vishnu P. Murty, Lauren A. Hudak, Jose L. Pascual, Mark J. Seamon, Elizabeth M. Datner, Claire Pearson, David A. Peak, Robert M. Domeier, Niels K. Rathlev, Brian J. O’Neil, Paulina Sergot, Leon D. Sanchez, Jutta Joormann, Diego A. Pizzagalli, Steven E. Harte, Ronald C. Kessler, Karestan C. Koenen, Kerry J. Ressler, Samuel A. McLean, Nathaniel G. Harnett

**Affiliations:** 1 Department of Psychiatry, Harvard Medical School, Boston, Massachusetts; 2 Division of Depression and Anxiety Disorders, McLean Hospital, Belmont, Massachusetts; 3 Department of Psychiatry and Behavioral Sciences, Emory University School of Medicine, Atlanta, Georgia; 4 Department of Psychological Sciences, University of Missouri-St Louis, St Louis; 5 Department of Emergency Medicine, Washington University School of Medicine, St Louis, Missouri; 6 Department of Epidemiology, Brown University, Providence, Rhode Island; 7 Department of Emergency Medicine, Brown University, Providence, Rhode Island; 8 Institute for Trauma Recovery, Department of Anesthesiology, University of North Carolina at Chapel Hill, Chapel Hill; 9 Departments of Psychiatry and Neurology, University of California San Francisco, San Francisco; 10 Department of Biomedical Informatics, Emory University School of Medicine, Atlanta, Georgia; 11 Department of Biomedical Engineering, Georgia Institute of Technology and Emory University, Atlanta; 12 Institute for Technology in Psychiatry, McLean Hospital, Belmont, Massachusetts; 13 The Many Brains Project, Belmont, Massachusetts; 14 Department of Psychology and Neuroscience, University of North Carolina at Chapel Hill, Chapel Hill; 15 Department of Sociology, University of North Carolina at Chapel Hill, Chapel Hill; 16 Department of Psychiatry, McLean Hospital, Belmont, Massachusetts; 17 Department of Emergency Medicine, University of Massachusetts Chan Medical School, Worcester; 18 Department of Emergency Medicine, Vanderbilt University Medical Center, Nashville, Tennessee; 19 Department of Emergency Medicine, Henry Ford Health System, Detroit, Michigan; 20 Department of Emergency Medicine, Indiana University School of Medicine, Indianapolis; 21 Department of Emergency Medicine, University of Florida College of Medicine -Jacksonville, Jacksonville; 22 Department of Emergency Medicine, Cooper Medical School of Rowan University, Camden, New Jersey; 23 Department of Emergency Medicine, Ohio State University College of Medicine, Columbus; 24 Ohio State University College of Nursing, Columbus; 25 Department of Emergency Medicine, Oakland University William Beaumont School of Medicine, Rochester, Michigan; 26 Department of Psychology, Temple University, Philadelphia, Pennsylvania; 27 Department of Emergency Medicine, Emory University School of Medicine, Atlanta, Georgia; 28 Department of Surgery, Department of Neurosurgery, University of Pennsylvania, Philadelphia; 29 Perelman School of Medicine, University of Pennsylvania, Philadelphia; 30 Department of Surgery, Division of Traumatology, Surgical Critical Care and Emergency Surgery, University of Pennsylvania, Philadelphia; 31 Department of Emergency Medicine, Jefferson Einstein Hospital, Jefferson Health, Philadelphia, Pennsylvania; 32 Department of Emergency Medicine, Sidney Kimmel Medical College, Thomas Jefferson University, Philadelphia, Pennsylvania; 33 Department of Emergency Medicine, Wayne State University, Ascension St John Hospital, Detroit, Michigan; 34 Department of Emergency Medicine, Massachusetts General Hospital, Boston; 35 Department of Emergency Medicine, Trinity Health-Ann Arbor, Ypsilanti, Michigan; 36 Department of Emergency Medicine, University of Massachusetts Medical School-Baystate, Springfield; 37 Department of Emergency Medicine, Wayne State University, Detroit Receiving Hospital, Detroit, Michigan; 38 Department of Emergency Medicine, McGovern Medical School at UTHealth, Houston, Texas; 39 Department of Emergency Medicine, Brigham and Women’s Hospital, Boston, Massachusetts; 40 Department of Emergency Medicine, Harvard Medical School, Boston, Massachusetts; 41 Department of Psychology, Yale University, New Haven, Connecticut; 42 Department of Anesthesiology, University of Michigan Medical School, Ann Arbor; 43 Department of Internal Medicine-Rheumatology, University of Michigan Medical School, Ann Arbor; 44 Department of Health Care Policy, Harvard Medical School, Boston, Massachusetts; 45 Department of Epidemiology, Harvard T.H. Chan School of Public Health, Harvard University, Boston, Massachusetts; 46 Department of Emergency Medicine, University of North Carolina at Chapel Hill, Chapel Hill; 47 Institute for Trauma Recovery, Department of Psychiatry, University of North Carolina at Chapel Hill, Chapel Hill

## Abstract

**Importance:**

Research on resilience after trauma has often focused on individual-level factors (eg, ability to cope with adversity) and overlooked influential neighborhood-level factors that may help mitigate the development of posttraumatic stress disorder (PTSD).

**Objective:**

To investigate whether an interaction between residential greenspace and self-reported individual resources was associated with a resilient PTSD trajectory (ie, low/no symptoms) and to test if the association between greenspace and PTSD trajectory was mediated by neural reactivity to reward.

**Design, Setting, and Participants:**

As part of a longitudinal cohort study, trauma survivors were recruited from emergency departments across the US. Two weeks after trauma, a subset of participants underwent functional magnetic resonance imaging during a monetary reward task. Study data were analyzed from January to November 2023.

**Exposures:**

Residential greenspace within a 100-m buffer of each participant’s home address was derived from satellite imagery and quantified using the Normalized Difference Vegetation Index and perceived individual resources measured by the Connor-Davidson Resilience Scale (CD-RISC).

**Main Outcome and Measures:**

PTSD symptom severity measured at 2 weeks, 8 weeks, 3 months, and 6 months after trauma. Neural responses to monetary reward in reward-related regions (ie, amygdala, nucleus accumbens, orbitofrontal cortex) was a secondary outcome. Covariates included both geocoded (eg, area deprivation index) and self-reported characteristics (eg, childhood maltreatment, income).

**Results:**

In 2597 trauma survivors (mean [SD] age, 36.5 [13.4] years; 1637 female [63%]; 1304 non-Hispanic Black [50.2%], 289 Hispanic [11.1%], 901 non-Hispanic White [34.7%], 93 non-Hispanic other race [3.6%], and 10 missing/unreported [0.4%]), 6 PTSD trajectories (resilient, nonremitting high, nonremitting moderate, slow recovery, rapid recovery, delayed) were identified through latent-class mixed-effect modeling. Multinominal logistic regressions revealed that for individuals with higher CD-RISC scores, greenspace was associated with a greater likelihood of assignment in a resilient trajectory compared with nonremitting high (Wald *z* test = −3.92; *P* < .001), nonremitting moderate (Wald *z* test = −2.24; *P* = .03), or slow recovery (Wald *z* test = −2.27; *P* = .02) classes. Greenspace was also associated with greater neural reactivity to reward in the amygdala (n = 288; *t*_277_ = 2.83; adjusted *P* value = 0.02); however, reward reactivity did not differ by PTSD trajectory.

**Conclusions and Relevance:**

In this cohort study, greenspace and self-reported individual resources were significantly associated with PTSD trajectories. These findings suggest that factors at multiple ecological levels may contribute to the likelihood of resiliency to PTSD after trauma.

## Key Points

**Question:**

Is there an association between residential greenspace/perceived individual resources and posttraumatic stress disorder (PTSD) trajectories after trauma?

**Findings:**

In this longitudinal cohort study of 2597 recent trauma survivors in the US, geocoded and self-reported variables were associated with different posttraumatic stress disorder (PTSD) trajectories. In individuals reporting higher individual resources, a greater neighborhood resource (residential greenspace) was associated with an increased likelihood of assignment in a resilient trajectory compared with a nonremitting high, nonremitting moderate, or slow recovery trajectory.

**Meaning:**

Results suggest that individual and neighborhood factors were associated with psychological outcomes after trauma; interactions between factors at different ecological levels are important in understanding the likelihood of resiliency to PTSD after trauma.

## Teaser

This cohort study investigates the association between neighborhood and individual resources and posttraumatic stress disorder trajectories after trauma.

## Introduction

Each year, over 46 million people experience a trauma requiring medical attention, and approximately 10% to 20% will develop posttraumatic stress disorder (PTSD).^1^ Previous efforts to differentiate trauma survivors who will be resilient vs those who develop PTSD may have been hindered, in part, because of an emphasis on individual-level factors without consideration of key neighborhood-level factors. Indeed, ecological frameworks propose multiple levels of influence on mental health, from individual-level resources such as psychological or cognitive abilities to cope with stress to neighborhood-level resources such as greenspace.^2^ Characterizing the effect of neighborhood-level factors on PTSD development may improve the early identification of individuals most at risk for the disorder and our understanding of resiliency to PTSD after trauma.

Resilience in the context of trauma often refers to low or no symptoms after a traumatic event (ie, a resilient trajectory), an outcome influenced by both dynamic processes and factors that increase the likelihood of resiliency (ie, resilience factors).^3^ Neighborhoods may provide a restorative environment that confers additional benefits beyond individual-level resilience factors or enhances individual-level factors.^4,5^ For example, greenspace is associated with lower levels of stress, anxiety, and depression, even after adjusting for individual factors including socioeconomic status (^6,7,8,9^ reviewed in^10^). In nearly 1 million individuals, childhood exposure to greenspace was associated with a lower risk of adulthood psychiatric disorders even after adjusting for parental history, socioeconomic factors, and urbanicity.^11^ Notably, greenspace represents a complex socioenvironmental factor that may be associated with mental health through various pathways, such as buffering against harmful environmental exposures and supporting health-promotion behaviors (eg, exercise) or psychological restoration and mindfulness.^12,13,14^

In individuals exposed to trauma, greenspace was associated with less severe PTSD and trauma-related distress.^9^ Greenspace attenuated the relationship between potentially traumatic events and general health in a sample of over 4500 individuals, even after adjusting for socioeconomic position and urbanicity.^15^ Further, greenspace was associated with lower anxiety and depression via a greater capacity to cope with stress in trauma-naive college students.^6^ Although additional work is needed to understand the mechanisms underlying the relationship between greenspace and PTSD development, these studies suggest that greenspace could be an important factor in resiliency to PTSD after trauma.

Factors that may increase the likelihood of resiliency to trauma are theorized to both dampen neurobiological stress-related mechanisms and activate reward-related circuitry; however, the latter is relatively understudied.^16,17^ Individuals with PTSD often exhibit altered neural reward processing, including decreased activation in regions involved in processing rewards when exposed to monetary reward, including the nucleus accumbens, amygdala, and orbitofrontal cortex (OFC).^18^ The nucleus accumbens is crucial for reinforcement learning and processes initial information about reward value and prediction error.^19^ The OFC is involved in processing reward value and reward-related decision-making whereas the amygdala underlies encoding reward-related information, updating reward value, and coordinating approach behaviors.^19^ Several studies^16,20,21^ have documented that activation and altered resting-state connectivity of these regions are associated with self-reported individual resources, such as self-efficacy and perceived ability to cope with adversity. Neuroimaging work on greenspace has focused on threat-related mechanisms.^22^ For example, acute exposure to a natural environment (via a 90-minute walk) is associated with decreased self-reported stress and diminished amygdala threat reactivity.^23^ Together, the emerging work suggests that the relationship between greenspace and PTSD development may be partly explained by differences in underlying neural reactivity.

In the present study, we merged existing data from a large US-based study on trauma^1^ with geospatial analytic techniques to evaluate whether greenspace was associated with PTSD trajectories after considering other self-reported and geocoded information. Based on previous work,^6^ we also tested whether there was a significant association between greenspace and perceived individual resources.^6^ We expected that greenspace would strengthen the association between individual resources and assignment in a recovery or resilient trajectory. As a secondary aim, we evaluated whether reward reactivity helped explain any associations between greenspace and trajectories. We expected greenspace would be associated with greater reward reactivity in the amygdala, nucleus accumbens, and the OFC and that greater reactivity would be related to assignment in a resilient trajectory.

## Methods

### Participants

Trauma survivors were recruited between September 2017 and June 2021 from emergency departments (EDs) within 72 hours of a traumatic injury.^1^ Complete details of the larger study (the Advancing Understanding of Recovery After Trauma [AURORA] study) are reported elsewhere.^1,24^ Procedures were approved by each site’s institutional review board. Individuals provided written informed consent and were financially compensated for their participation. Exclusion and inclusion criteria are presented in the eMethods in Supplement 1. Approximately 2 weeks after trauma, a subset of participants underwent neuroimaging.^1,24,25^ Scanning was conducted at 2 weeks to help facilitate the early detection of neural markers of PTSD development.^1^ This study followed the Strengthening the Reporting of Observational Studies in Epidemiology (STROBE) reporting guidelines.

### Measures

#### Demographics and Injury Assessment

In the ED, participants self-reported their sex at birth, age, marital status, and ethnoracial group (race and ethnicity were queried separately and later merged into a single variable). Study participants self-identified with the following races and ethnicities: non-Hispanic Black, Hispanic, non-Hispanic White, and non-Hispanic other race, which included American Indian, Asian, Pacific Islander, and other. Injury characteristics, including physician-evaluated Injury Severity Scores (ISS) and self-reported head injury were recorded in the ED. At the 2-week visit, participants reported their annual household income, which was transformed into a semi-continuous variable such that every 1-unit increase corresponded to an additional $20 000 to $25 000 per year.

#### Psychometric Assessments

At 2 weeks after trauma, the 10-item Connor-Davidson Resilience Scale (CD-RISC) was administered to measure perceived individual resources.^26^ Participants rated how accurately each of the statements (eg, “I am able to adapt to change”) described them on a scale of 0 (not true at all) to 4 (true nearly all the time).^27^ Childhood maltreatment and lifetime trauma were evaluated using 5-items of the 11-item Childhood Trauma Questionnaire–Short Form^28^ and the Life Events Checklist for *DSM-5 *(LEC-5),^29^ respectively. The PTSD Symptom Checklist for *DSM-5* (PCL-5) was administered at the 2-week, 8-week, 3-month, and 6-month visits, and evaluated the presence and severity of symptoms.^26^ Participants rated the severity of the 20-items on a scale of 0 (not at all) to 4 (extremely).^26^ Additional details on assessments, including metrics of internal reliability, are provided in the eMethods in Supplement 1.

#### Neighborhood-Level Factors

##### Residential Greenspace

High-resolution (30-m) multiband satellite imagery from the Landsat 8 archive was extracted from Google Earth Engine^30,31^ (eMethods and eFigure 2 in Supplement 1). Within ArcGIS Pro, version 3.0.0 (ESRI), a 100-m Euclidean buffer was created around each address as prior work revealed this size buffer shows peak associations with mental health outcomes.^32^ Zonal spatial analyses were conducted to extract the mean Normalized Difference Vegetation Index (NDVI) values within each buffer.

##### Neighborhood Socioeconomic Disadvantage

Participants’ home addresses were matched to the corresponding Area Deprivation Index (ADI), version 3.1 2019.^33,34,35^ The ADI is available online^36^ and is a weighted composite measure of neighborhood disadvantage that considers 17 census items spanning domains such as employment, income, and housing quality.

### Magnetic Resonance Imaging Acquisition and Analysis

Neuroimaging data were collected across 5 sites with harmonized acquisition protocols on Siemens 3-T magnetic resonance imaging (MRI) scanners (eTable 1 in Supplement 1). As part of the modified card guessing game,^24^ participants viewed cards with a question mark (2 seconds) before guessing whether the card’s value was higher or lower than 5 (values randomly varied from 0-9). After a delay (2-4 seconds), the card’s value and monetary outcome were displayed. Before the task, participants were informed that they would win $1 for each correct guess and lose $0.50 for each incorrect guess. A total of 40 cards were presented (20 gains and 20 losses).

Preprocessing was performed using fMRIPrep, version 1.2.2 (open source) as reported in previous work^24^ (eMethods in Supplement 1). Gain and loss trials were modeled as separate events convolved with a canonical hemodynamic response function. “Gain* > *loss” was the contrast of interest. The mean across all voxels in each bilateral region of interest (ROI) was extracted from first-level contrasts and activity was averaged across hemispheres.

### Statistical Analysis

All analyses were completed in R, version 4.1.2 (R Project for Statistical Computing). First, latent-class mixed-effect modeling was conducted using the hlme function in the lcmm package.^37^ Participants who completed the PCL-5 for at least 2 of the 4 time points (2 week, 8 week, 3 month, and 6 month) were included Based on previous work, we compared 1 to 7 classes and selected the best model based on entropy, Bayesian information criterion (BIC), Akaike information criterion, sample-size adjusted Bayesian information criterion, and log-likelihood reductions.^38^ Based on recommendations for reporting latent-class mixed-effect models, BIC and entropy were favored.^39,40,41^ We also considered the average posterior probabilities to determine how certain the model was at distinguishing the class for each participant (<0.70 is recommended).^38^ Finally, theoretical basis and parsimony were weighted heavily when determining the best model. At least 1% of participants were required to be assigned to a class, and classes were required to be interpretable based on previous work.^38^ We further compared our approach, which allowed for nonlinear trajectories, with previously reported linear trajectories (analyses conducted using Mplus) from our group (eTable 2 in Supplement 1).^42,43^

Next, multinominal logistic regressions (multinom package) were conducted to evaluate the associations between self-reported and geospatially derived measures and PTSD trajectories. Continuous measures were grand mean-centered across the full sample. The mice package was used to handle missing data, which were imputed using predictive mean matching with 20 imputations. None of the variables in the main analyses had more than 10% missingness. The resilient group was set as the reference; therefore, the model included one contrast testing how variables contributed to the odds of falling into a specific trajectory compared to the resilient trajectory. We tested whether NDVI and CD-RISC were independently associated with trajectories after adjusting for age, income, ADI ranking, ISS, marital status (0 = unmarried), head injury (0 = did not hit head), LEC-5 score, and childhood maltreatment. These variables were selected as covariates based on previous work suggesting they contribute to PTSD trajectories in ED-recruited samples.^39,44^ Our primary model examined whether an NDVI × CD-RISC interaction was prospectively associated with trajectory assignment. Wald *z* tests were used to examine the significance of each individual coefficient.

To evaluate whether neural reward reactivity was a possible pathway by which greenspace or CD-RISC was associated with a resilient trajectory, we first conducted general linear models (GLMs) to determine whether these factors were associated with responses in the amygdala, nucleus accumbens, and OFC after covarying for income, ADI ranking, sex, age, marital status, ISS, LEC-5 score, and childhood maltreatment. These 3 ROIs were tested because of their established roles in reward-related processing, their relationship with PTSD symptom severity in the AURORA study,^24^ and to limit exploratory analyses and reduce the number of multiple-comparison corrections required. After identifying significant ROI(s), a 1-way analysis of variance (ANOVA) was conducted to determine whether reactivity was significantly different across the trajectories. Holm-Bonferroni correction was applied to each set of GLMs (eg, 3 tests examining the association of NDVI with the ROIs,) and a corrected α level of .05 was used for all statistical tests. A *P* value of <.05 was considered significant, and all *P* values were 2-sided. Study data were analyzed from January to November 2023.

## Results

### Sample Characteristics

A total of 2597 trauma survivors (mean [SD] age, 36.5 [13.4]; 1637 female [63%]; 960 male [37.0%]; 1304 non-Hispanic Black [50.2%], 289 Hispanic [11.1%], 901 non-Hispanic White [34.7%], 93 non-Hispanic other [3.6%], and 10 missing/unreported [0.4%]) were included in this analysis. eFigure 1 in Supplement 1 depicts the study flowchart. Demographic characteristics are presented in Table 1. Correlations between continuous self-report and geocoded variables are presented in eTable 3 in Supplement 1.

**Table 1.** Sample Characteristics

Variable	No. (%)	
	Full sample (N = 2597)	MRI subsample (n = 288)
Sex at birth		
Female	1637 (63.0)	185 (64.2)
Male	960 (37.0)	103 (35.8)
Age, mean (SD), y	36.5 (13.4)	34.7 (13.0)
Ethnoracial group		
Non-Hispanic Black	1304 (50.2)	124 (43.1)
Hispanic	289 (11.1)	43 (14.9)
Non-Hispanic White	901 (34.7)	106 (36.8)
Non-Hispanic other^a^	93 (3.6)	13 (4.5)
Missing	10 (0.4)	2 (0.7)
Income		
<$19 000	793 (30.5)	88 (30.6)
$19 001-35 000	749 (28.8)	88 (30.6)
$35 001-50 000	331 (12.7)	37 (12.8)
$50 001-75 000	201 (7.7)	27 (9.4)
$75 001-100 000	165 (6.4)	19 (6.6)
>$100 000	182 (7.0)	29 (10.1)
Missing	176 (6.8)	0
Marital status		
Married	552 (21.3)	48 (16.7)
Unmarried	2031 (78.2)	240 (83.3)
Missing	14 (0.5)	0
Injury Severity Score, mean (SD) [missing]	2.4 (1.9) [1]	2.4 (1.9)
Childhood maltreatment, mean (SD) [missing]	9.4 (9.8) [264]	10.2 (10.6)
CD-RISC score, mean (SD) [missing]	22.6 (8.1) [138]	22.5 (7.3)
Normalized Vegetation Difference Index, mean (SD) [missing]	0.5 (0.1) [0]	0.4 (0.2)
Area Deprivation Index, mean (SD) [missing]	64.4 (27.7) [84]	56.9 (28.9)
Week 2 PTSD symptoms (PCL-5 scores), mean (SD) [missing]^b^	31.1 (18.9) [232]	28.5 (16.7)
Week 8 PTSD symptoms (PCL-5 scores), mean (SD) [missing]^b^	28.0 (19.5) [197]	26.1 (17.6)
Month 3 PTSD symptoms (PCL-5 scores), mean (SD) [missing]^b^	25.1 (19.2) [334]	23.2 (17.9)
Month 6 PTSD symptoms (PCL-5 scores), mean (SD) [missing]^b^	23.3 (18.7) [660]	21.1 (18.2)

Abbreviations: CD-RISC, Connor-Davidson Resilience Scale; MRI, magnetic resonance imaging; PCL-5, PTSD Symptom Checklist for *DSM-5*; PTSD, posttraumatic stress disorder.

^a^ Other includes American Indian, Asian, Pacific Islander, and other.

^b^ Participants were required to have completed PCL-5 at least twice (full sample: 232 missing week 2; 197 missing week 8; 334 missing month 3; 660 missing month 6; MRI sample: 0 missing week 2; 25 missing week 8; 39 missing month 3; 57 missing month 6).

### Identification and Prospective Associations with PTSD Trajectories

**Figure 1.**
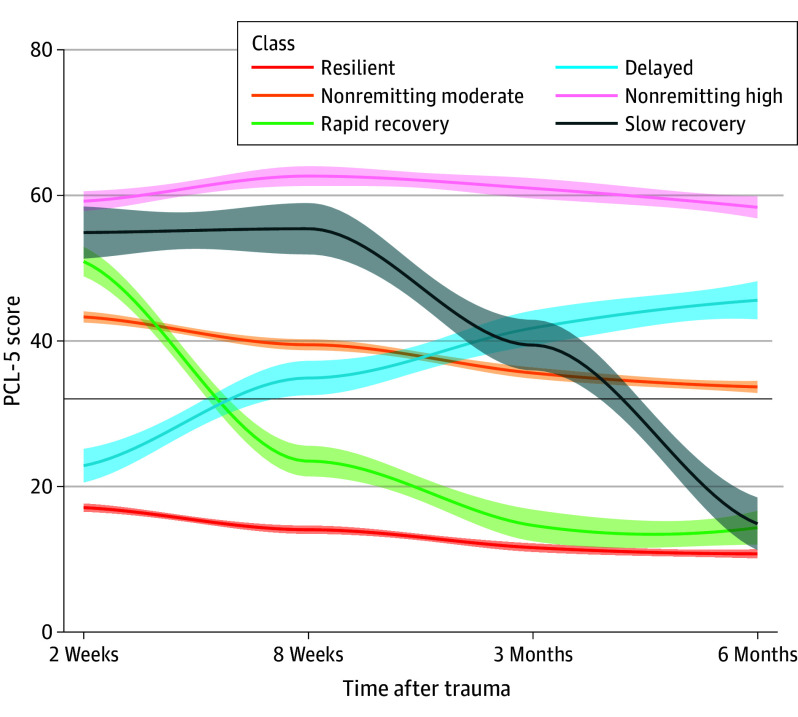
Results of Latent-Class Mixed-Effect Modeling The largest classes were the resilient (1318 [50.8%]), nonremitting moderate (734 [28.3%]), and nonremitting high (244 [9.4%]) trajectories, whereas the smallest classes were the delayed (108 [4.2%]), slow recovery (67 [2.6%]) and the rapid recovery (126 [4.9%]) groups. The solid black line represents the clinically significant cutoff for the Posttraumatic Stress Disorder Symptom Checklist for *DSM-5* (total score = 32).

Latent-class mixed-effect models revealed a 6-group solution with a linear and quadratic term for time was the best fit to the data (Figure 1 and eTable 4 [for fit indices] and eTable 5 [for fit indices with linear term] in Supplement 1). Plots for all fitted models (eFigure 3 in Supplement 1), details on model selection (average posterior probabilities in eTable 6 in Supplement 1), and characterization of the 6 identified trajectories (resilient, nonremitting high, nonremitting moderate, delayed, rapid recovery, and slow recovery) are provided in eMethods in Supplement 1. Group differences (pairwise comparisons with Holm-Bonferroni correction applied) between trajectories on both self-reported and geocoded measures were also noted (eTable 7 in Supplement 1). There was no difference between classes on NDVI; however, CD-RISC scores were significantly higher in the resilient trajectory (mean [SD], 24.14 [8.42]) vs the rapid recovery (mean [SD], 21.31 [6.93]; *P* adjusted = .001), delayed (mean [SD], 21.96 [7.35]; *P* adjusted = .04), and moderate nonremitting trajectories (mean [SD], 21.25 [6.35];* P *adjusted < .001). Further, individuals in the nonremitting high group (mean [SD], 1.83 [8.20]) had significantly lower CD-RISC scores compared with individuals in the resilient (*P* adjusted < .001), rapid recover (*P* adjusted = .04), delayed (*P* adjusted = .005), and nonremitting moderate groups (*P* adjusted < .001).

The logistic regression identified self-reported and geocoded variables that were associated with symptomatic class memberships compared with the resilient trajectory (eTable 8 in Supplement 1 for results without interaction term). NDVI was not related to class assignments. Higher CD-RISC scores significantly increased the likelihood of assignment in the resilient trajectory compared with a nonremitting high (Wald *z* test = −7.96; *P* < .001), nonremitting moderate (Wald *z* test = −6.51; *P* < .001), delayed (Wald *z* test = −2.49; *P* = .01), and rapid recovery (Wald *z* test = −2.91; *P* = .004) classes.

**Figure 2.**
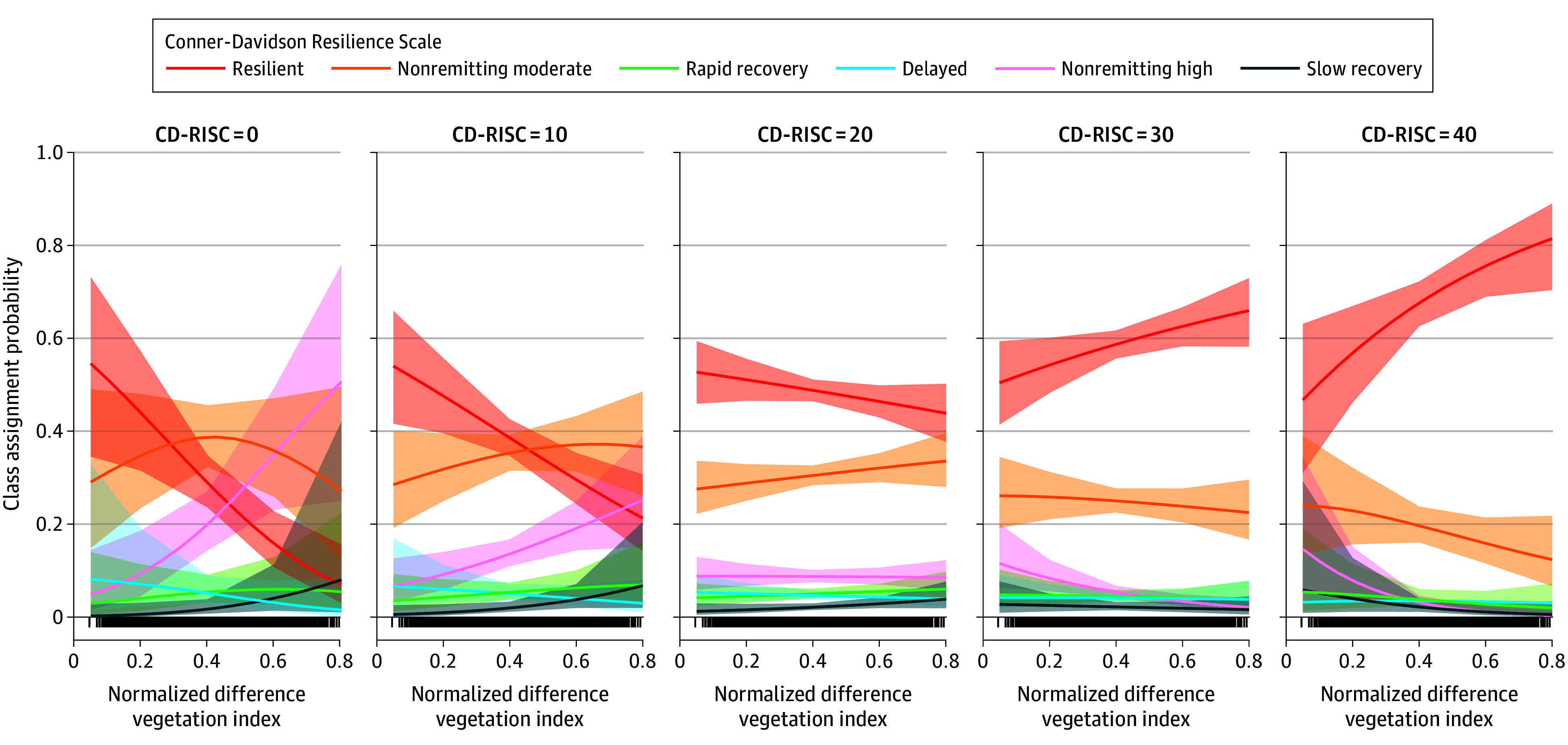
Association Between Neighborhood and Individual Resources and Posttraumatic Stress Disorder Trajectory Assignment There was a significant association between greenspace and Connor-Davidson Resilience Scale (CD-RISC) scores on class assignment, such that individuals reporting higher levels of perceived internal resources with higher residential greenspace had an even greater likelihood of assignment in the resilient trajectory compared with the nonremitting high, nonremitting moderate, and slow recovery classes.

The primary model (Table 2) revealed that at higher scores of CD-RISC, higher NDVI was associated with increased likelihood of assignment in the resilient trajectory compared with the nonremitting high (Wald *z* test = −3.92; *P* < .001), nonremitting moderate (Wald *z* test = −2.24; *P* = .03), or slow recovery (Wald *z* test = −2.27; *P* = .02) classes even after considering the other variables (Figure 2). Details about significant covariates are provided in the eMethods in Supplement 1. A sensitivity analysis (eTable 9 in the Supplement) revealed covarying for baseline PTSD and medication use, only the significant interaction in the nonremitting high group compared with the resilient group remained (Wald *z* test = −3.09; *P* = .002), and there was no effect for the nonremitting moderate or slow-recovery groups. Further, an exploratory model examining income × CD-RISC scores was conducted (eTable 10 and eFigure 4 in Supplement 1).

**Table 2.** Self-Report and Geocoded Variables Associated With Class Membership (Full Sample)^a^

Variable	Trajectory class (statistical tests relative to the resilient trajectory)														
	High nonremitting			Moderate nonremitting			Delayed			Slow recovery			Rapid recovery		
	Coefficient (SE)	Wald *z*	*P* value	Coefficient (SE)	Wald *z*	*P* value	Coefficient (SE)	Wald *z*	*P* value	Coefficient (SE)	Wald *z*	*P* value	Coefficient (SE)	Wald *z*	*P* value
Intercept	−2.32 (0.17)	−13.51	<.001	−1.08 (0.11)	−10.02	<.001	−2.40 (0.20)	−11.96	<.001	−3.57 (0.30)	−11.95	<.001	−2.61 (0.21)	−12.58	<.001
Sex at birth (male)^b^	0.36 (0.16)^c^	2.28^c^	.02^c^	0.54 (0.10)^c^	5.17^c^	<.001^c^	−0.07 (0.21)	−0.34	.73	0.34 (0.27)	1.25	.21	0.38 (0.20)	1.86	.06
CD-RISC	−0.08 (0.01)^c^	−8.27^c^	<.001^c^	−0.04 (0.01)^c^	−6.64^c^	<.001^c^	−0.03 (0.01)^c^	−2.47^c^	.01^c^	−0.03 (0.02)	−1.72	.09	−0.04 (0.01)^c^	−3.00^c^	.003^c^
NDVI	−0.52 (0.58)	−0.93	.35	0.24 (0.36)	0.60	.55	−0.24 (0.74)	−0.35	.72	1.04 (0.99)	1.02	.31	0.39 (0.70)	0.55	.55
ISS	0 (0.04)	0.09	.93	0 (0.03)	0.16	.86	0.01 (0.05)	0.24	.81	0.12 (0.06)^c^	2.12^c^	.03^c^	0.09 (0.05)^c^	2.07^c^	.04^c^
Age	0.01 (0.01)^c^	2.18^c^	.03^c^	0.01 (0)^c^	3.34^c^	.001^c^	0.01 (0.01)	1.15	.25	−0.01 (0.01)	−0.56	.58	−0.02 (0.01)^c^	−2.00^c^	.046^c^
Income	−0.20 (0.06)^c^	−3.16^c^	.002^c^	−0.11 (0.04)^c^	−2.79^c^	.005^c^	0.04 (0.07)	0.60	.55	−0.26 (0.12)^c^	−2.21^c^	.03^c^	−0.08 (0.07)	−1.10	.27
ADI ranking	0	1.40	.16	0	1.52	.13	0	0.19	.85	0.01 (0.01)^c^	1.99^c^	.046^c^	0	−0.07	.94
Marital status (unmarried)	−0.29 (0.21)	−1.33	.18	−0.01 (0.13)	−0.06	.95	−0.17 (0.27)	−0.64	.52	−0.02 (0.36)	−0.05	.96	−0.30 (0.29)	−1.05	.29
Childhood maltreatment	0.07 (0.01)^c^	9.53^c^	<.001^c^	0.05 (0.01)^c^	9.21^c^	<.001^c^	0.05 (0.01)^c^	4.59^c^	<.001^c^	0.06 (0.01)^c^	5.24^c^	<.001^c^	0.05 (0.01)^c^	4.75^c^	<.001^c^
Head injury (did not hit head)	0.43 (0.15)^c^	2.80^c^	.005^c^	0.34 (0.10)^c^	3.36^c^	.001^c^	0.09 (0.21)	0.42	.68	0.45 (0.27)	1.67	.10	0.19 (0.20)	0.96	.34
LEC-5	0.05 (0.01)^c^	6.18^c^	<.001^c^	0.03 (0.01)^c^	6.10^c^	<.001^c^	0.01 (0.01)	1.04	.30	0.03 (0.01)^c^	2.17^c^	.03^c^	0.01 (0.01)	0.89	.38
NDVI × CD-RISC	−0.29 (0.07)^c^	−3.92^c^	<.001^c^	−0.11 (0.05)^c^	−2.24^c^	.03^c^	−0.03 (0.09)	−0.36	.72	−0.29 (0.13)^c^	−2.27^c^	.02^c^	−0.14 (0.09)	−1.55	.12

Abbreviations: ADI, Area Deprivation Index (national ranking); CD-RISC, Connor-Davidson Resilience Scale (total score); ISS, Injury Severity Score; LEC-5, Life Events Checklist for *DSM-5* (total score); NDVI, Normalized Difference Vegetation Index.

^a^ Continuous measures were grand-mean centered in the full sample.

^b^ The reference group for dichotomous variables is provided in parentheses.

^c^ Numbers correspond to uncorrected *P* < .05.

### Greenspace, Neural Responses to Reward, and PTSD

**Figure 3.**
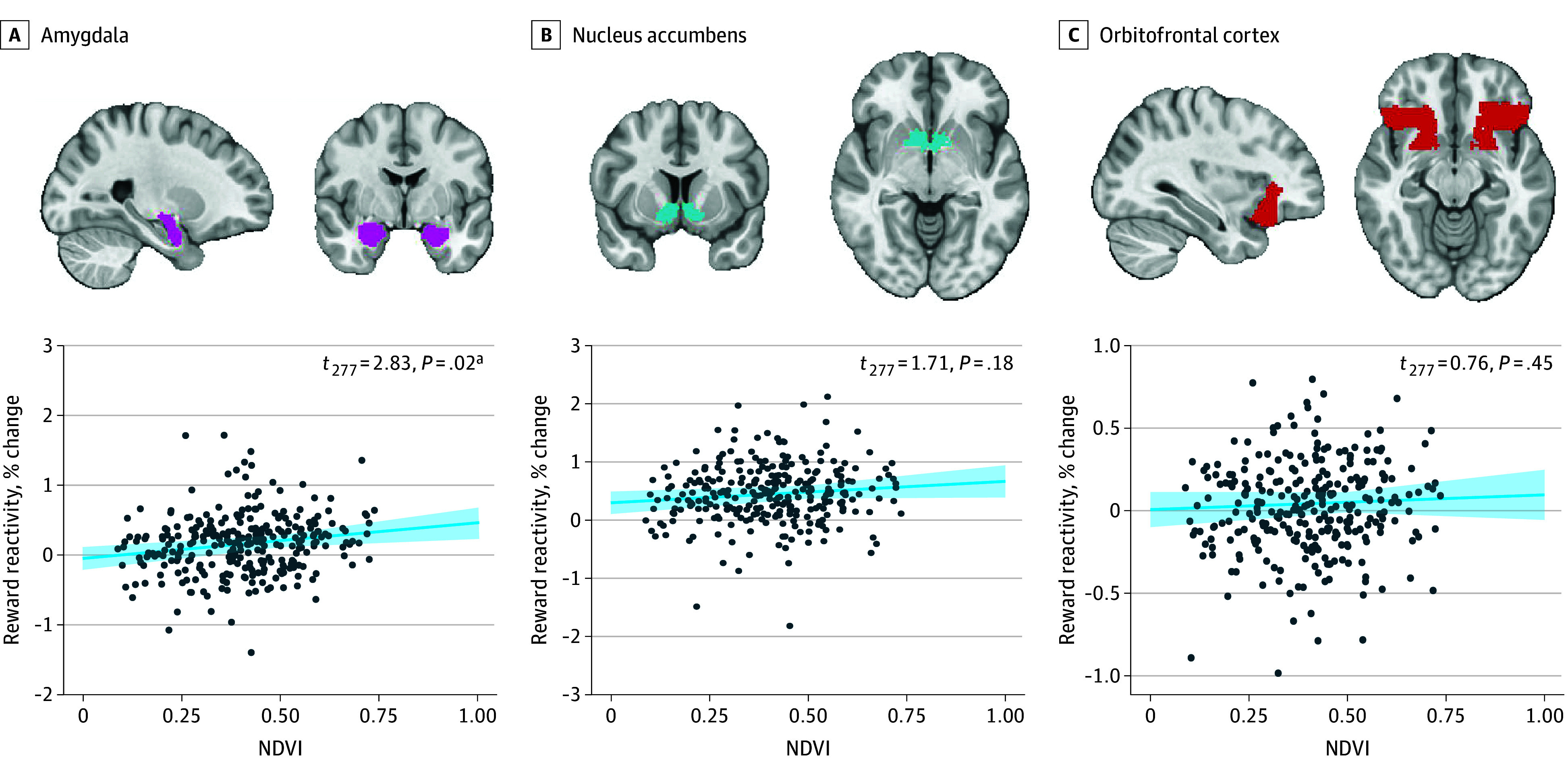
Greenspace and Neural Responses to Reward Greater residential greenspace was associated with neural responses to reward in the amygdala (A) but not in the nucleus accumbens (B) or orbitofrontal cortex (C) after adjusting for sex at birth, Connor-Davidson Resilience Scale, Injury Severity Score, age, income, area deprivation index, marital status, Life Events Checklist for *DSM-5*, and childhood maltreatment. These are marginal effects plots depicting predicted values of neural responses across normalized difference vegetation index (NDVI) values (shaded line: 95% CIs for the marginal effects; data points: observed data; *P* values are Holm-Bonferroni adjusted. ^a^Adjusted *P* < .05.

The results of the logistic regression in the MRI sample are presented in eTable 11 in Supplement 1). GLMs (eTable 12 in Supplement 1) revealed higher NDVI was associated with greater reactivity within the amygdala (n = 288; *t*_277_ = 2.83; β = 0.18; adjusted *P *= .02) (Figure 3A) after adjusting for covariates. There was no significant main effect of CD-RISC on amygdala reactivity. NDVI was not associated with reward responses in the nucleus accumbens (*t*_277_ = 1.71; β = 0.11; adjusted *P* = 0.18) (Figure 3B), or OFC (*t*_277_ = 0.76; β = 0.05; adjusted *P* = 0.45) (Figure 3C). Finally, there were no significant associations between NDVI and CD-RISC on reactivity (eTable 13 in Supplement 1).

A one-way ANOVA revealed that amygdala reactivity was not significantly different by trajectory groups (*F*_4,283_ = 0.36; *P* = .84). Therefore, additional analyses testing whether greenspace was associated with trajectory assignment via amygdala reactivity were not conducted. Nucleus accumbens and OFC reactivity did not significantly differ by trajectory group (eMethods in Supplement 1).

## Discussion

In this cohort study, we identified factors at multiple ecological levels that were prospectively associated with PTSD risk and resilience after trauma. We characterized 6 PTSD trajectories and identified a novel interaction between greenspace and CD-RISC scores in 3 classes (nonremitting high, nonremitting moderate, and rapid recovery). The majority of work on resilience factors and PTSD examines whether individual-level factors moderate the link between trauma exposure and symptoms. For example, self-reported internal resources and social support buffer against the impact of traumatic events on PTSD symptoms.^45,46,47^ However, an individual’s response to trauma occurs in the context of their environment and may be shaped by neighborhood influences. Our findings suggest that quantifying greenspace is relevant to understanding both PTSD trajectories and reward reactivity in recent trauma survivors.

Greenspace alone was not associated with a resilient trajectory, nor was it independently associated with any of the other trajectories. There are 2 possible explanations as to how individual and neighborhood resources may interact which warrant future work. First, individual resources, such as the ability to think clearly under pressure, may be necessary to access the possible protective features of urban green space. Second, greenspace may support the development, maintenance, and expansion of an individual’s capacity to cope with stress.^48^ For example, individuals living in more advantaged neighborhoods with more access to greenspace may be faced with isolated stressors as opposed to chronic life stress. An individual’s perception of their individual-level resources may be reinforced when they overcome a single event and may wane when faced with unrelenting adversity.^49^ Previous work supports both explanations, highlighting that neighborhood and individual factors dynamically interact to support resiliency across the lifespan.^50,51^

Although the contribution of the amygdala to aversive learning is well-known, this region also plays a role in determining the value of stimuli, forming cue-reward associations, and coordinating approach behavior.^52^ Greenspace is associated with reduced threat-related amygdala activity^22^; however, the present study was, to our knowledge, the first to show an association between greenspace and reward reactivity. One possible pathway by which greenspace is associated with amygdala reactivity to reward may be through increased attentional capacity to identify stimuli as rewarding. The Attention Restoration Theory^53^ suggests exposure to greenspace reduces attentional demands and ultimately replenishes cognitive resources required to attend to stimuli. Greater attentional capacity may facilitate amygdala activity while updating reward values during the task, although future work is required to directly test this pathway.

In contrast to our hypotheses, amygdala reactivity was not associated with PTSD trajectories. Previous work suggests trauma exposure may change how the amygdala responds to reward. For example, amygdala responses to happy vs neutral faces and gains vs losses are significantly lower in participants with PTSD and depression.^54^ Earlier work from the AURORA study found participants with low reactivity to reward and high threat reactivity were more likely to have more severe PTSD symptoms.^24^ In general, greater activation of reward circuitry is related to better trauma outcomes^55^; however, our findings suggest that the association between greenspace and reward circuitry may not be the pathway supporting resilience to PTSD after trauma.

One explanation is that we examined PTSD trajectories, operationalizing resilience as low or no PTSD symptoms, rather than examining transdiagnostic markers such as anhedonia (ie, inability to experience pleasure) or rumination (ie, negative repetitive thoughts). Anhedonia is a dimension of both PTSD and depression and is consistently associated with neural responsivity to reward.^56,57^ Following a 90-minute walk in a natural setting, individuals exhibited both decreased rumination symptoms and lower resting-state activity in the subgenual prefrontal cortex, which plays a role in self-referential thought. Future work on greenspace may benefit by using a transdiagnostic dimensional approach and/or defining posttraumatic resilience as the absence of any form of posttraumatic dysfunction.

### Limitations

This study has some limitations. The study recruitment sites were predominantly in urban areas of the Midwest, South, and Northeast, precluding an examination of the moderating effect of urbanicity.^58^ Assumptions about the intrinsic therapeutic value offer little space for regional differences and individual preferences. Relatedly, individuals may change residence and have varying levels of greenspace throughout their lifespan. Unfortunately, we did not query the participant’s residential history or record address changes across the study. Future directions may include investigating how various aspects of natural infrastructure and different terrains impact mental health with a keen focus on time-varying and moderating effects. In addition, we did not capture information regarding greenspace use. Therefore, this study cannot conclude whether the observed effects reflect any use, passive engagement, or active use.^59,60^ Cross-sectional work has suggested greenspace is associated with more physical activity.^61^ Future work should consider examining objective measures of physical activity, greenspace, and PTSD in trauma survivors.

Finally, CD-RISC may be capturing other constructs such as positive emotionality (disposition to experience positive emotions),^62^ and conflating both traitlike characteristics and dynamic changes in self-reported resilience.^63^ Bonanno^64^ suggested that single assessments of self-reported resilience such as the CD-RISC often fail to predict PTSD outcomes because they do not capture resilience as a flexible process that is sensitive to context and temporal dynamics. Indeed, an individual’s perception of their ability to cope may be influenced by their PTSD symptoms as well as other factors (eg, emotion regulation strategy preference) not measured in this study.

## Conclusions

Results of this cohort study have important implications for the clinical care of trauma survivors and trauma-informed policy efforts. For example, efforts to improve access and quality of urban greenspace may benefit the millions of individuals exposed to trauma each year. In addition, perceptions of individual resources, which can be targeted through individual interventions (eg, cognitive behavioral therapy) may be further enhanced by greenspace exposure. In conclusion, this study adds to the emerging evidence that disentangling heterogeneity in trauma outcomes requires consideration of factors at multiple ecological levels.

## Notes

Supplement 1.
eMethods.

eReferences.
**eTable 1.** Harmonized MRI Sequences Across Study Sites **eTable 2.** Overlap Between New and Previously Reported Trajectories **eTable 3.** Pearson Correlations Between Study Measures in the Full and MRI Sample **eTable 4.** Fit Indices for the Latent-Class Mixed-Effect Models (Nonlinear Solutions) **eTable 5.** Fit Indices for Latent-Class Mixed Modeling Analysis (Linear Solutions) **eTable 6.** Average Posterior Probabilities for Nonlinear Solution 6-Class Solution **eTable 7.** Characteristics by Assigned Trajectory **eTable 8.** Self-Report and Geocoded Variables Associated With Class Membership (Full Sample; No Interaction Term) **eTable 9.** Self-Report and Geocoded Variables Associated With Class Membership (Full Sample; Interaction Term; Adjusting for Baseline PTSD and Medication Use) **eTable 10.** Self-Report and Geocoded Variables Associated With Class Membership (Full Sample; With Income × CD-RISC Interaction) **eTable 11.** Self-Report and Geocoded Variables Associated With Class Membership (MRI Sample; With NDVI × CD-RISC Interaction) **eTable 12.** General Linear Models for Reward Reactivity (Main Effect of NDVI) **eTable 13.** General Linear Models for Reward Reactivity (With NDVI × CD-RISC Interaction) **eFigure 1.** Study Flowchart **eFigure 2.** Normalized Difference Vegetation Index Around MRI Study Sites **eFigure 3.** Results of the Latent-Class Mixed-Effect Models (1 To 7 Classes) **eFigure 4.** Interaction Between Individual-Level Factors (CD-RISC × Income) on Trajectory Assignment

Supplement 2.
Data Sharing Statement.
