# Supplementary material for: Neighborhood Resources Associated With Psychological Trajectories and Neural Reactivity to Reward After Trauma
Source: JAMA Psychiatry. 2024 Jul 31;81(11):1090–100. doi: 10.1001/jamapsychiatry.2024.2148 (PMC11292566; doi:10.1001/jamapsychiatry.2024.2148)
Supplement: Supplement 1. — eMethods. eReferences. eTable 1. Harmonized MRI Sequences Across Study Sites eTable 2. Overlap Between New and Previously Reported Trajectories eTable 3. Pearson Correlations Between Study Measures in the Full and MRI Sample eTable 4. Fit Indices for the Latent-Class Mixed-Effect Models (Nonlinear Solutions) eTable 5. Fit Indices for Latent-Class Mixed Modeling Analysis (Linear Solutions) eTable 6. Average Posterior Probabilities for Nonlinear Solution 6-Class Solution eTable 7. Characteristics by Assigned Trajectory eTable 8. Self-Report and Geocoded Variables Associated With Class Membership (Full Sample; No Interaction Term) eTable 9. Self-Report and Geocoded Variables Associated With Class Membership (Full Sample; Interaction Term; Adjusting for Baseline PTSD and Medication Use) eTable 10. Self-Report and Geocoded Variables Associated With Class Membership (Full Sample; With Income × CD-RISC Interaction) eTable 11. Self-Report and Geocoded Variables Associated With Class Membership (MRI Sample; With NDVI × CD-RISC Interaction) eTable 12. General Linear Models for Reward Reactivity (Main Effect of NDVI) eTable 13. General Linear Models for Reward Reactivity (With NDVI × CD-RISC Interaction) eFigure 1. Study Flowchart eFigure 2. Normalized Difference Vegetation Index Around MRI Study Sites eFigure 3. Results of the Latent-Class Mixed-Effect Models (1 To 7 Classes) eFigure 4. Interaction Between Individual-Level Factors (CD-RISC × Income) on Trajectory Assignment [file jamapsychiatry-e242148-s001.pdf]

Supplementary Online Content

Webb EK, Stevens JS, Ely TD, et al. Neighborhood resources associated with psychological trajectories and neural reactivity to reward after trauma. *JAMA Psychiatry*. Published online July 31, 2024. doi:10.1001/jamapsychiatry.2024.2148

eMethods.

eReferences.

eTable 1. Harmonized MRI Sequences Across Study Sites

eTable 2. Overlap Between New and Previously Reported Trajectories

eTable 3. Pearson Correlations Between Study Measures in the Full and MRI Sample

eTable 4. Fit Indices for the Latent-Class Mixed-Effect Models (Nonlinear Solutions)

eTable 5. Fit Indices for Latent-Class Mixed Modeling Analysis (Linear Solutions)

eTable 6. Average Posterior Probabilities for Nonlinear Solution 6-Class Solution

eTable 7. Characteristics by Assigned Trajectory

eTable 8. Self-Report and Geocoded Variables Associated With Class Membership (Full Sample; No Interaction Term)

eTable 9. Self-Report and Geocoded Variables Associated With Class Membership (Full Sample; Interaction Term; Adjusting for Baseline PTSD and Medication Use)

eTable 10. Self-Report and Geocoded Variables Associated With Class Membership (Full Sample; With Income × CD-RISC Interaction)

eTable 11. Self-Report and Geocoded Variables Associated With Class Membership (MRI Sample; With NDVI × CD-RISC Interaction)

eTable 12. General Linear Models for Reward Reactivity (Main Effect of NDVI)

eTable 13. General Linear Models for Reward Reactivity (With NDVI × CD-RISC Interaction)

eFigure 1. Study Flowchart

eFigure 2. Normalized Difference Vegetation Index Around MRI Study Sites

eFigure 3. Results of the Latent-Class Mixed-Effect Models (1 To 7 Classes)

eFigure 4. Interaction Between Individual-Level Factors (CD-RISC × Income) on Trajectory Assignment

This supplementary material has been provided by the authors to give readers additional information about their work.

© 2024 Webb EK et al. *JAMA Psychiatry.*

1. Supplementary Methods

1.1. Satellite Imagery Preprocessing.

To obtain the highest quality data (i.e., during summer months) and evaluate greenspace immediately before the study period, we acquired satellite images between May 1st, 2017, through September 30th, 2017. Images with less than 20% cloud cover were considered useable. In Google Earth Engine (1,2), images underwent preprocessing steps including atmospheric correction and Top of Atmosphere reflectance conversion to remove effects from water vapor and sun position. Computation of the normalized difference vegetation index (NDVI) was performed in GEE with the following equation: NDVI = (near-infrared band – red band / near-infrared band + red band) (3). Raw NDVI values ranged from -1 to 1, with greater values representing denser vegetation and lower numbers reflecting snow or water. The NDVI rasters and the coordinates of the participants' home addresses were then entered into ArcGIS Pro Version 3.0.0 (ESRI, 2018) for further processing. To avoid penalizing natural infrastructure, bodies of water were masked out (i.e., set as empty values). As part of this process, final NDVI values were transformed to range from 0 to 1 (see **eFigure 2**).

1.2. Participants - Inclusion/Exclusion Criteria.

Individuals were eligible for the study if they were between 18-75 years old, able to read and write in English, and alert/oriented. Qualifying traumatic events included motor vehicle collisions, physical or sexual assaults, falls greater than 10 feet, or an experience that otherwise met *Diagnostic and Statistical Manual of Mental Disorders 5th edition* (DSM-5) criterion A for PTSD (4) and that the research team agreed that was a plausible qualifying event. Participants were excluded if they: sustained a solid organ injury greater than grade 1 or had a significant

© 2024 Webb EK et al. *JAMA Psychiatry.*

hemorrhage, were intubated, required general anesthesia, or were likely to be admitted for more than three days.

Additional exclusion criteria applied for the neuroimaging portion of the study (see supplement), including the presence of metal or ferromagnetic material in the body, claustrophobia, a history of neurodegenerative disorders, or a history of seizures.

1.3. MRI Preprocessing in fMRIPrep

Results included in this manuscript come from preprocessing performed using fMRIPrep v1.2.2 (5,6). **eTable2** describes the harmonized MRI acquisition parameters across each site. Each T1w (T1-weighted) volume was corrected for INU (intensity non-uniformity) using N4BiasFieldCorrection v2.1.0 (7) and skull-stripped using antsBrainExtraction.sh v2.1.0 (using the OASIS template). Brain surfaces were reconstructed using recon-all from FreeSurfer v6.0.1, and the brain mask estimated previously was refined with a custom variation of the method to reconcile ANTs-derived and FreeSurfer-derived segmentations of the cortical gray-matter of Mindboggle (8).

Spatial normalization to the ICBM 152 Nonlinear Asymmetrical template version 2009c (9) was performed through nonlinear registration with the antsRegistration tool of ANTs v2.1.0 (10), using brain-extracted versions of both T1w volume and template. Brain tissue segmentation of cerebrospinal fluid (CSF), white-matter (WM) and gray-matter (GM) was performed on the brain-extracted T1w using fast (FSL v5.0.9). Functional data was slice-time corrected using 3dTshift from AFNI v16.2.07 and motion corrected using mcflirt (FSL v5.0.9). This was followed by co-registration to the corresponding T1w using boundary-based registration with six degrees of freedom, using bbregister (FreeSurfer v6.0.1). Motion correcting transformations,

© 2024 Webb EK et al. *JAMA Psychiatry.*

BOLD-to-T1w transformation and T1w-to-template (MNI) warp were concatenated and applied in a single step using antsApplyTransforms (ANTs v2.1.0) using Lanczos interpolation.

Frame-wise displacement was calculated for each functional run using the implementation of Nipype. ICA-based Automatic Removal Of Motion Artifacts (AROMA) was used to generate aggressive noise regressors as well as to create a variant of data that is non-aggressively denoised (11). For more details of the pipeline see https://fmriprep.readthedocs.io/en/stable/workflows.html. An overall motion threshold was also implemented such that any participant's task data with >15% volumes and ≥1-mm framewise displacement were excluded.

In first-level analyses, gain and loss trials were modeled as separate events convolved with a canonical hemodynamic response function. *Gain* > *loss* was the contrast for the region of interest extraction (ROI). ROIs were selected based on previous work (12) and defined anatomically using the Automated Anatomical Atlas (12). Reward ROIs included the nucleus accumbens, OFC, and amygdala.

1.4. Psychometric Assessments.

At two-weeks post-trauma, the 10-item Connor-Davidson Resilience Scale (CD-RISC) was administered to measure perceived individual resources (13). The CD-RISC demonstrates good internal reliability (α= .85) (14). Childhood maltreatment was evaluated using 5-items of the 11-item Childhood Trauma Questionnaire-Short Form (15). These items were selected to both sufficiently assess childhood maltreatment and minimize participant burden (16). Together, these items have been shown to have high reliability (α= .92) (16). Participants reported how often they experienced each of the items, which captured both abuse and neglect, using a 5-point Likert scale (from 0: *never* to 4: *very often*). The total score was the sum of all the items.

© 2024 Webb EK et al. *JAMA Psychiatry.*

Lifetime trauma exposure to 17 potentially traumatic/stressful events was evaluated using the Life Events Checklist for DSM-5 (17). Participants indicated whether they had experienced, witnessed, or learned about the event. A total score was created by summing all responses, with higher numbers indicating a more extensive trauma history. The LEC-5 has excellent internal consistency (α = .91).

The PTSD Symptom Checklist for DSM-5 (PCL-5) was administered at the 2-week, 8-week, 3-month, and 6-month study visit, and evaluated the presence and severity of various posttraumatic stress symptoms (13). Prior work indicates the PCL-5 has excellent internal consistency (α = .94) and test-retest reliability (r = .82) (18).

1.5. Selection and Characterization of PTSD trajectories

The six-group solution (resilient, nonremitting high, nonremitting moderate, slow recovery, rapid recovery, and delayed trajectory) with a linear and quadratic term for time was selected as the best fit for the data. Although entropy was slightly higher in the five-class solution (5-class entropy = .72 vs. 6-class entropy = .71), BIC, AIC, SABIC, and log-likelihood were lower in the six-class solution. The 7-class solution had the lowest BIC, AIC, SABIC, and reductions in log-likelihood but the lowest entropy (.69). In addition, the 7-class solution lacked parsimony and theoretical basis, identifying two similar nonremitting moderate classes. Posterior probabilities (all > .70) suggested that individuals were more likely to be classified into their assigned group compared to an alternative group indicating reasonable accuracy of the six-class model (reported in **eTable6**). Therefore, we selected the six-group solution over the 7-group solution to ensure the interpretability of the classes.

The six symptom classes corresponded with a resilient (low symptoms across time), nonremitting high (symptoms far exceed the clinically relevant cut-off of PCL-5 total score of 32

© 2024 Webb EK et al. *JAMA Psychiatry.*

across time), nonremitting moderate (symptoms slightly above the clinically relevant cut-off across time), slow recovery (elevated symptoms slowly decreasing across time), rapid recovery (elevated symptoms decreasing to below the PCL-5 cut-off score by 8-weeks post-trauma), and delayed trajectory (increasing symptoms across time).

Prior investigations reveal that the majority of trauma survivors experience no or low symptoms (resilient trajectory) whereas a subset is highly symptomatic (nonremitting trajectory). While resilient and nonremitting classes frequently emerge, additional trajectories including nonremitting moderate, rapid recovery, delayed, and slow recovery, may also emerge (19,20). Differences between the number and type of trajectories identified may be influenced by sample characteristics. For example, Tomas et al., (2022) compared two ED samples (admitted and discharged) and identified a unique delayed class in the hospitalized sample which was not present in individuals who were discharged. Another study identified 5 trajectories (resilient, nonremitting/chronic, recovery, delayed, and worsening/recovery) among 9/11 police responders and a 6-class solution among 9/11 non-traditional responders (resilient, recovering, delayed, chronic, moderate-low, and nonremitting moderate) (21). While the trajectories identified in the current analysis align well with prior work, future work directly examining differences in trajectories between samples (e.g., interpersonal violence vs. motor vehicle vs. war-related events) is warranted.

Significant differences (pairwise comparisons with Holm-Bonferroni correction applied) between the trajectories are presented in **eTable7**. There was no significant difference between the rapid and slow recovery groups on 2-week PCL-5 symptoms; however, at 8 weeks, there was a significant difference between all the groups. The 3-month PCL-5 scores did not differ between the delayed vs. the slow recovery or between the slow recovery vs. the moderate group. Finally,

© 2024 Webb EK et al. *JAMA Psychiatry.*

at 6 months post-injury, there was no significant difference between the rapid and slow recovery groups, with both groups exhibiting low symptoms (but still elevated compared to the resilient group). ADI was significantly higher (reflective of greater neighborhood disadvantage) in the nonremitting high, nonremitting moderate, and slow recovery groups compared to the resilient group.

Income was significantly lower in the rapid recovery vs. the resilient trajectory, and lower in the nonremitting high vs resilient and delayed trajectories. Income was also significantly lower in the slow recovery and nonremitting moderate classes compared to the resilient class. Individuals in the rapid recovery trajectory were significantly younger than those assigned to the resilient and nonremitting moderate trajectories. Individuals in resilient trajectory had lower lifetime trauma history (LEC-5 scores) compared to individuals in the nonremitting high and moderate trajectories whereas those in the rapid recovery class had lower trauma load than the nonremitting high class. There was a significant difference in childhood maltreatment between the resilient trajectory (lower CTQ scores) and all other trajectories. The rapid recovery group had significantly lower CTQ scores compared to the nonremitting high group. In addition, the nonremitting high group was exposed to greater childhood maltreatment compared to the delayed group.

1.6. Covariates and PTSD Trajectories

Beyond the NDVI x CD-RISC term of interest, there were several significant predictors of trajectories in the full model (presented in the main text **Table 3**). Females were more likely to be assigned either of the nonremitting high (Wald's $z = 2.28$, $p = .02$) and nonremitting moderate classes (Wald's $z = 5.17$, $p < .001$) versus a resilient class. Higher ISS was associated with an increased likelihood of a slow recovery (Wald's $z = 2.12$, $p = .03$) or rapid recovery (Wald's $z =$

© 2024 Webb EK et al. *JAMA Psychiatry.*

2.07, $p = .04$) trajectory compared to a resilient trajectory. ADI was associated with assignment in the slow recovery group versus the resilient group (Wald's $z = 1.99$, $p = .046$). Lower income was also associated with an increased likelihood of falling into a nonremitting high (Wald's $z = -3.16$, $p = .002$), nonremitting moderate (Wald's $z = -2.79$, $p = .005$), or slow recovery class (Wald's $z = -2.21$, $p = .03$) compared to a resilient class.

In line with prior work, childhood maltreatment increased the likelihood of assignment in the nonremitting high (Wald's $z = 9.53$, $p < .001$), nonremitting moderate (Wald's $z = 9.21$, $p < .001$), delayed (Wald's $z = 4.59$, $p < .001$), slow recovery (Wald's $z = 5.24$, $p < .001$), and rapid recovery (Wald's $z = 4.75$, $p < .001$) trajectories compared to a resilient trajectory. Lifetime trauma increased the likelihood of assignment in the nonremitting high (Wald's $z = 6.18$, $p < .001$), nonremitting moderate (Wald's $z = 6.10$, $p < .001$), and slow recovery (Wald's $z = 2.17$, $p = .03$) groups compared to the resilient group. Finally, a head injury/hitting head during the traumatic event increased the likelihood of assignment in the nonremitting high (Wald's $z = 2.80$, $p = .005$) or nonremitting moderate (Wald's $z = 3.36$, $p = .001$) groups compared to the resilient group. Taken together these results align well with prior work finding that lower income, older age, female sex, and greater childhood maltreatment are associated with more severe and chronic PTSD (19,22–24). Our work also aligns with other ED-recruited trauma survivors which suggests more severe injuries (as reflected by higher ISS or head injury) can result in more severe PTSD symptoms (19,23). However, analyses of the injury characteristics were limited to two variables, and further research in this area is needed (e.g. examining the effect of objective measures of traumatic brain injury).

1.7. Sensitivity Analysis with Baseline PTSD Symptoms and Medication Use

We examined whether the NDVI x CD-RISC interaction in the full sample held after adjusting for baseline/pre-existing PTSD symptoms and medication use (0: *no medication;* as reported at 2 weeks post-injury; included selective serotonin reuptake inhibitors and/or serotonin and norepinephrine reuptake inhibitors, and/or benzodiazepines). Only 1722 individuals (66.3%) completed a PCL-5 querying their PTSD symptoms in the 30 days prior to the traumatic event (n = 841 screened positive). Only 1398 (53.8%) completed the medication screen (n = 291 screened positive). Even when these variables (imputed with the *mice* package using predictive mean matching with 20 imputations) were included in the multinominal logistic regression, individuals with higher CD-RISC scores who had greater greenspace exposure had an increased likelihood of assignment in a resilient trajectory compared to a nonremitting high trajectory (Wald's z = -3.09, p = .002; **eTable9**).

1.8 Interaction Between Individual-level Factors

We also tested whether an Income x CD-RISC interaction was present in the full sample after adjusting for covariates (**eTable10**). This analysis revealed that at higher scores of CD-RISC, greater annual household income was associated with increased likelihood of assignment in the resilient trajectory compared to the nonremitting high (Wald z test = -5.73; $p < .001$), nonremitting moderate (Wald z test = -4.58; $p < .001$), delayed (Wald z test = -3.06; p = .002, rapid recovery (Wald z test = -2.89; p = .004), or slow recovery classes (Wald z test = -3.75, $p < .001$) even after considering the other variables (depicted in **eFigure4**).

1.9 Prediction of PTSD Trajectories in MRI Sample

A multinomial logistic regression model predicting PTSD trajectories (as assigned from the full sample trajectory analyses) was conducted in the MRI subset sample (**eTable11**). However, the rapid recovery (n = 4) and slow recovery classes (n = 13) were combined into a

© 2024 Webb EK et al. *JAMA Psychiatry.*

single recovery group to ensure sufficient sample sizes in each class. Thus, there were 5 classes: resilient (n = 165), nonremitting moderate (n = 71), recovery (n = 17), delayed (n = 17) and nonremitting high (n = 18). There was no significant interaction between CD-RISC scores and NDVI ($ps > .05$).

1.10. ROI Reward Reactivity Between Classes

One-way ANOVAs revealed that nucleus accumbens and OFC reactivity to reward did not differ by PTSD trajectory classes (nucleus accumbens: $F(4,283) = 0.54$, $p = .704$; OFC: $F(4,283) = 1.01$, $p = .405$).

© 2024 Webb EK et al. *JAMA Psychiatry.*

eReferences

1. Helbich M, Poppe R, Oberski D, Zeylmans van Emmichoven M, Schram R (2021): Can't see the wood for the trees? An assessment of street view- and satellite-derived greenness measures in relation to mental health. *Landsc Urban Plan* 214: 104181.

2. Burrows K, Fong KC, Lowe SR, Fussell E, Bell ML (2023): The impact of residential greenness on psychological distress among Hurricane Katrina survivors. *PLOS ONE* 18: e0285510.

3. Measuring Vegetation (NDVI & EVI) [Text.Article] (2000, August 30): NASA Earth Observatory. Retrieved June 13, 2023, from https://earthobservatory.nasa.gov/features/MeasuringVegetation

4. American Psychiatric Association AP, Association AP (2013): Diagnostic and statistical manual of mental disorders: DSM-5.

5. Esteban O, Blair R, Markiewicz CJ, Berleant SL, Moodie C, Ma F, Gorgolewski KJ (2017): poldracklab/fmriprep: 1.0. 0-rc5. *Geneva Zenedo*.

6. Esteban O, Markiewicz CJ, Blair RW, Moodie CA, Isik AI, Erramuzpe A, *et al.* (2019): fMRIPrep: a robust preprocessing pipeline for functional MRI. *Nat Methods* 16: 111–116.

7. Tustison NJ, Avants BB, Cook PA, Zheng Y, Egan A, Yushkevich PA, Gee JC (2010): N4ITK: improved N3 bias correction. *IEEE Trans Med Imaging* 29: 1310–1320.

8. Fischl B (2012): FreeSurfer. *Neuroimage* 62: 774–781.

9. Fonov V, Evans AC, Botteron K, Almli CR, McKinstry RC, Collins DL, Brain Development Cooperative Group (2011): Unbiased average age-appropriate atlases for pediatric studies. *NeuroImage* 54: 313–327.

© 2024 Webb EK et al. *JAMA Psychiatry.*

10. Avants BB, Tustison N, Johnson H (n.d.): Advanced Normalization Tools (ANTS).

11. Pruim RHR, Mennes M, van Rooij D, Llera A, Buitelaar JK, Beckmann CF (2015): ICA-AROMA: A robust ICA-based strategy for removing motion artifacts from fMRI data. *NeuroImage* 112: 267–277.

12. Stevens JS, Harnett NG, Lebois LAM, van Rooij SJH, Ely TD, Roeckner A, *et al.* (2021): Brain-Based Biotypes of Psychiatric Vulnerability in the Acute Aftermath of Trauma. *Am J Psychiatry* 178: 1037–1049.

13. Weathers FW, Litz BT, Keane TM, Palmieri PA, Marx BP, Schnurr PP (2013): The ptsd checklist for dsm-5 (pcl-5). *Scale Available Natl Cent PTSD Www Ptsd Va Gov* 10: 206.

14. Campbell-Sills L, Stein MB (2007): Psychometric analysis and refinement of the Connor-davidson Resilience Scale (CD-RISC): Validation of a 10-item measure of resilience. *J Trauma Stress* 20: 1019–1028.

15. Bernstein DP, Stein JA, Newcomb MD, Walker E, Pogge D, Ahluvalia T, *et al.* (2003): Development and validation of a brief screening version of the Childhood Trauma Questionnaire. *Child Abuse Negl* 27: 169–190.

16. Wong SA, Lebois LAM, Ely TD, van Rooij SJH, Bruce SE, Murty VP, *et al.* (2023): Internal capsule microstructure mediates the relationship between childhood maltreatment and PTSD following adulthood trauma exposure. *Mol Psychiatry* 1–10.

17. Weathers FW, Blake DD, Schnurr PP, Kaloupek DG, Marx BP, Keane TM (2013): The life events checklist for DSM-5 (LEC-5).

18. Blevins CA, Weathers FW, Davis MT, Witte TK, Domino JL (2015): The Posttraumatic Stress Disorder Checklist for DSM-5 (PCL-5): Development and Initial Psychometric Evaluation. *J Trauma Stress* 28: 489–498.

© 2024 Webb EK et al. *JAMA Psychiatry.*

19. Tomas CW, Fitzgerald JM, Bergner C, Hillard CJ, Larson CL, deRoon-Cassini TA (2022): Machine learning prediction of posttraumatic stress disorder trajectories following traumatic injury: Identification and validation in two independent samples. *J Trauma Stress* 35: 1656–1671.

20. Brier ZMF, Connor J, Legrand AC, Price M (2020): Different Trajectories of PTSD Symptoms During the Acute Post-Trauma Period. *J Psychiatr Res* 131: 127–131.

21. Pietrzak RH, Feder A, Singh R, Schechter CB, Bromet EJ, Katz CL, *et al.* (2014): Trajectories of PTSD risk and resilience in World Trade Center responders: an 8-year prospective cohort study. *Psychol Med* 44: 205–219.

22. Shansky RM (2015): Sex differences in PTSD resilience and susceptibility: Challenges for animal models of fear learning. *Neurobiol Stress* 1: 60–65.

23. deRoon-Cassini TA, Mancini AD, Rusch MD, Bonanno GA (2010): Psychopathology and resilience following traumatic injury: a latent growth mixture model analysis. *Rehabil Psychol* 55: 1–11.

24. Bryant RA, Nickerson A, Creamer M, O'Donnell M, Forbes D, Galatzer-Levy I, *et al.* (2015): Trajectory of post-traumatic stress following traumatic injury: 6-year follow-up. *Br J Psychiatry J Ment Sci* 206: 417–423.

25. Short NA, van Rooij SJH, Murty VP, Stevens JS, An X, Ji Y, *et al.* (2022): Anxiety sensitivity as a transdiagnostic risk factor for trajectories of adverse posttraumatic neuropsychiatric sequelae in the AURORA study. *J Psychiatr Res* 156: 45–54.

© 2024 Webb EK et al. *JAMA Psychiatry.*

eTable1. Harmonized MRI sequences across study sites

	**Site 1**	**Site 2**	**Site 3**	**Site 4**	**Site 5**
**Scanner**	Siemens TIM 3T Trio	Siemens TIM 3T Trio	Siemens MAGNETOM 3T Prisma	Siemens 3T Verio	Siemens MAGNETOM 3T Prisma
**Head Coil**	12 Channel	12 Channel	20 Channel	12 Channel	20 Channel
**Modality**					
**T1-weighted**	**TR** = 2530ms, **TEs** = 1.74/3.6/5.46/7.32ms, **TI** = 1260ms, **flip angle** = 7, **FOV** = 256mm, **slices** = 176, **Voxel size** = 1mm x 1mm x 1mm	**TR** = 2530ms, **TEs** = 1.74/3.6/5.46/7.32ms, **TI** = 1260ms, **flip angle** = 7, **FOV** = 256mm, **slices** = 176, **Voxel size** = 1mm x 1mm x 1mm	**TR** = 2300ms, **TE** = 2.96ms, **TI** = 900ms, **flip angle** = 9, **FOV** = 256mm, **slices** = 176, **Voxel size** = 1.2mm x 1.0mm x 12mm	**TR** = 2530ms, **TEs** = 1.74/3.65/5.51/7.72ms, **TI** = 1260ms, **flip angle** = 7, **FOV** = 256mm, **slices** = 176, **Voxel size** = 1mm x 1mm x 1mm	**TR** = 2300ms, **TE** = 2.98ms, **TI** = 900ms, **flip angle** = 9, **FOV** = 256mm, **slices** = 176, **Voxel size** = 1.2mm x 1.0mm x 12mm
**Diffusion Weighted Imaging**	**TR** = 7700ms, **TE** = 85ms, **FOV** = 212mm, **flip angle** = 90, **Volumes** = 71 (64 **b**=1000 s/mm^{2,} 7 b0), **PA-encoded**, **Voxel size** = 2mm x 2mm x 2mm	**TR** = 7700ms, **TE** = 85ms, **FOV** = 212mm, **flip angle** = 90, **Volumes** = 71 (64 **b**=1000 s/mm^{2,} 7 b0), **PA-encoded**, **Voxel size** = 2mm x 2mm x 2mm	**TR** = 7000ms, **TE** = 74ms, **FOV** = 212mm, **flip angle** = 90, **Volumes** = 71 (64 **b**=1000 s/mm^{2,} 7 b0), **PA-encoded**, **Voxel size** = 2mm x 2mm x 2mm	**TR** = 12000ms, **TE** = 85ms, **FOV** = 212mm, **flip angle** = 90, **Volumes** = 71 (64 **b**=1000 s/mm^{2,} 7 b0), **PA-encoded**, **Voxel size** = 2mm x 2mm x 2mm	**TR** = 7700ms, **TE** = 67ms, **FOV** = 212mm, **flip angle** = 90, **Volumes** = 71 (64 **b**=1000 s/mm^{2,} 7 b0), **PA-encoded**, **Voxel size** = 2mm x 2mm x 2mm
**fMRI**	**TR** = 2360ms, **TE** = 30ms, **flip angle** = 70, **FOV** = 212mm, **slices** = 44, **Voxel size** = 3mm x 2.72mm x 2.72mm, 0.5 mm gap	**TR** = 2360ms, **TE** = 30ms, **flip angle** = 70, **FOV** = 212mm, **slices** = 44, **Voxel size** = 3mm x 3mm x 3mm, 0.5 mm gap	**TR** = 2360ms, **TE** = 29ms, **flip angle** = 70, **FOV** = 212mm, **slices** = 44, **Voxel size** = 3mm x 2.72mm x 2.72mm, 0.5 mm gap	**TR** = 2360ms, **TE** = 30ms, **flip angle** = 70, **FOV** = 212mm, **slices** = 42, **Voxel size** = 3mm x 2.72mm x 2.72mm, 0.5 mm gap	**TR** = 2360ms, **TE** = 29ms, **flip angle** = 90, **FOV** = 210mm, **slices** = 44, **Voxel size** = 3mm x 3mm x 2.5mm, 0.5 mm gap

© 2024 Webb EK et al. *JAMA Psychiatry.*

eTable2. Overlap between new and previously reported trajectories

Prior linear trajectories*	New Non-linear Trajectories					
	Resilient	Nonremitting moderate	Rapid recovery	Delayed	Slow recovery	Nonremitting high
Low	**1252**	98	**86**	4	4	0
High - decreasing	0	15	22	0	**55**	0
Moderate	66	**609**	18	**85**	8	42
High	0	12	0	19	0	**202**

Note: There was considerable overlap between the previously reported 4 trajectories (see ref 25) and the 6 trajectories from the current approach. Numbers in bold represent overlap with the largest number of participants in each class.

© 2024 Webb EK et al. *JAMA Psychiatry.*

eTable3. Pearson's correlations between study measures in the full and MRI sample

Measure	NDVI	CD-RISC	ADI	Income	Childhood Maltreatment	ISS	Age	LEC-5
**Full Sample (N = 2,597)**								
NDVI								
CD-RISC	**.05***							
ADI	**-.08***	**-.06***						
Income	**.15***	**.15***	**-.36***					
Childhood Maltreatment	**-.07***	**-.18***	**.10***	**-.19***				
ISS	**.08***	.03	**-.07***	**.11***	**-.04***			
Age	**.08***	**.11***	**-.07***	**.17***	**-.07***	**.09***		
LEC-5	.01	**.04***	**-.07***	**.09***	**.15***	**.03**	**.09***	
**MRI Sample (*n* = 288)**								
NDVI								
CD-RISC	.03							
ADI	**-.24***	-.06						
Income	**.20***	**.18***	**-.24***					
Childhood Maltreatment	-.03	**-.25***	**.13***	**-.23***				
ISS	.04	.11	-.03	**.18***	-.06			
Age	**.14***	.06	**-.17***	**.21***	-.06	.11		
LEC-5	-.01	.02	**-.16***	.09	**.19***	<.01	.07	

Abbreviations: **ADI:** Area Deprivation Index (national ranking); **CD-RISC:** Connor-Davidson Resilience Scale (total score); **ISS***:* Injury Severity Score; **LEC-5:** Life Events Checklist for DSM-5 (total score); **PCL-5:** PTSD Checklist for DSM-5 (total symptom severity); **NDVI:** Normalized Difference Vegetation Index; *Notes:* correlations derived following mean imputation*; * p < .05.*

© 2024 Webb EK et al. *JAMA Psychiatry.*

eTable4. Fit indices for the latent class mixed effect models (non-linear solutions)

Class	*loglik*	*AIC*	*BIC*	*entropy*	*SABIC*	*%class1*	*%class2*	*%class3*	*%class4*	*%class5*	*%class6*	*%class7*
**1**	-36051.57	72117.15	72158.18	1.00	72135.94	100.00						
**2**	-35935.00	71892.00	71956.48	0.63	71921.53	66.92	33.08					
**3**	-35837.10	71704.21	71792.14	0.65	71744.48	9.90	63.53	26.57				
**4**	-35780.36	71598.72	71710.10	0.68	71649.73	9.97	23.30	3.23	63.50			
**5**	-35735.19	71516.38	71651.21	0.72	71578.13	23.64	9.51	1.54	62.30	3.00		
**6**	**-35696.96**	**71447.91**	**71606.19**	**0.71**	**71520.40**	**28.26**	**50.75**	**4.85**	**4.16**	**2.58**	**9.40**	
**7**	-35663.85	71389.70	71571.42	0.69	71472.93	51.06	17.71	11.47	4.24	2.43	2.96	10.13

Abbreviations: **Loglik**: log likelihood; **AIC:** Akaike information criterion**; BIC:** Bayesian information criterion**; SABIC:** Sample-size adjusted Bayesian information criterion.

© 2024 Webb EK et al. *JAMA Psychiatry.*

eTable5. Fit indices for latent class mixed modeling analysis (linear solutions)

Class	loglik	AIC	BIC	entropy	SABIC	%class1	%class2	%class3	%class4	%class5	%class6	%class7
**1**	-36057.05	72124.09	72153.41	1.00	72137.52	100.00						
**2**	-35952.84	71921.69	71968.58	0.63	71943.17	66.04	33.96					
**3**	-35881.39	71784.78	71849.26	0.65	71814.31	11.32	23.03	65.65				
**4**	-35837.53	71703.06	71785.13	0.72	71740.65	1.31	65.15	24.07	9.47			
**5**	-35802.14	71638.28	71737.94	0.71	71683.92	1.93	29.42	9.43	53.14	6.08		
**6**	-35784.01	71608.02	71725.26	0.71	71661.72	1.31	5.70	9.86	30.54	48.75	3.85	
**7**	-35770.79	71587.58	71722.41	0.69	71649.33	1.19	4.16	16.21	40.47	5.43	27.19	5.35

Abbreviations: **Loglik**: log likelihood; **AIC:** Akaike information criterion**; BIC:** Bayesian information criterion**; SABIC:** Sample-size adjusted Bayesian information criterion.

eTable6. Average posterior probabilities for non-linear solution 6-class solution

	Mean of posterior probabilities in each class					
Class	*prob1*	*prob2*	*prob3*	*prob4*	*prob5*	*prob6*
**1**	**0.70**	0.10	0.05	0.05	0.04	0.07
**2**	0.07	**0.88**	0.03	0.02	0.00	0.00
**3**	0.12	0.10	**0.74**	0.00	0.04	0.00
**4**	0.16	0.08	0.00	**0.70**	0.00	0.05
**5**	0.14	0.03	0.05	0.00	**0.76**	0.02
**6**	0.14	0.00	0.00	0.03	0.04	**0.79**

Note: Numbers in bold represent average posterior probability for classification in that class for observation classified in the specific class.

© 2024 Webb EK et al. *JAMA Psychiatry.*

eTable7. Trajectory characteristics

Variable	Assigned Trajectory					
	Rapid recovery (*n* = 126)	Nonremitting high (*n* = 244)	Resilient (*n* = 1318)	Delayed (*n* = 108)	Slow recovery (*n* = 67)	Nonremitting moderate (*n* = 734)
Sex at birth (% female and [*n*])	67.46 [85]	68.85 [168]	55.61 [753]	57.41 [62]	67.16 [45]	71.39 [524]
Hit head (% yes and [*n*])	57.93 [73]	60.65 [148]	49.47 [652]	52.78 [57]	64.18 [43]	57.63 [423]
Age in years (*Mean*)	32.60[a]	36.23	36.44[a]	37.15	33.94	37.44[a]
Income (*Mean*)	2.26[a]	1.98[b]	2.67[abc]	2.61[b]	1.93[c]	2.29[c]
Marital Status (% married and [*n*])	14.28 [*n* = 18]	15.16 [*n* = 37]	23.36 [*n* = 308]	21.29 [*n* = 23]	16.41 [*n* = 11]	21.25 [*n* = 156]
ISS (*Mean*)	2.67	2.36	2.42	2.45	2.81	2.39
Childhood Maltreatment (*Mean*)	11.33[a]	15.33[ab]	6.64[abc]	10.89[bc]	14.00[c]	12.50[bc]
LEC-5 (*Mean*)	8.33[b]	11.83[ab]	7.75[a]	8.92	9.87	10.52[a]
CD-RISC score (*Mean*)	21.31[a]	18.83[b]	24.14[abc]	21.96[b]	21.64	21.25[bc]
NDVI (*Mean*)	0.45	0.43	0.45	0.44	0.45	0.44
ADI (*Mean*)	64.84	68.75[a]	62.26[ab]	63.36	73.27[b]	66.41[b]
WK2 PCL-5 scores (*Mean*)	50.90[a]	58.83[ab]	17.35[abc]	22.94[abcd]	54.33[bcde]	42.62[abcde]
WK8 PCL-5 scores (*Mean*)	23.52*	62.41*	14.08*	34.61*	55.21*	39.33*
M3 PCL-5 scores (*Mean*)	15.39[a]	60.27[ab]	11.80[abc]	41.67[abcd]	39.30[abcd]	35.57[abcd]
M6 PCL-5 scores (*Mean*)	14.79[a]	57.55[ab]	10.79[abc]	43.66[abcd]	17.21[bcde]	33.22[abcde]

Abbreviations: **ADI:** Area Deprivation Index (national ranking); **CD-RISC:** Connor-Davidson Resilience Scale (total score); **ISS**: Injury Severity Score; **LEC-5:** Life Events Checklist for DSM-5 (total score); **PCL-5:** PTSD Checklist for DSM-5 (total symptom severity); **NDVI:** Normalized Difference Vegetation Index. *Notes:* Significant differences between trajectories that withstood Holm-Bonferroni correction for multiple comparisons are noted with row-level superscript letters ([a-d]). * notes all trajectories were significantly different.

© 2024 Webb EK et al. *JAMA Psychiatry.*

eTable8. Self-report and geocoded variables associated with class membership (full sample; no interaction term)

Variable	Nonremitting High				Nonremitting Moderate				Delayed				Slow recovery				Rapid recovery			
	**Trajectory Class** *(statistical tests relative to the resilient trajectory)*																			
	Coefficient	*SE*	*Wald Z*	*p-value*	*Coefficient*	*SE*	*Wald Z*	*p-value*	*Coefficient*	*SE*	*Wald Z*	*p-value*	*Coefficient*	*SE*	*Wald Z*	*p-value*	*Coefficient*	*SE*	*Wald Z*	*p-value*
Intercept	-2.29	0.17	-13.46	<.001	-1.08	0.11	-10.07	<.001	-2.40	0.20	-11.98	<.001	-3.56	0.30	-11.95	<.001	-2.61	0.21	-12.60	<.001
Sex at Birth [*male*]	0.34	0.16	2.15	.032	0.53	0.10	5.14	<.001	-0.07	0.21	-0.35	.728	0.32	0.27	1.16	.248	0.37	0.20	1.83	.067
CD-RISC	-0.08	0.01	-7.96	<.001	-0.04	0.01	-6.51	<.001	-0.03	0.01	-2.49	.013	-0.03	0.02	-1.66	.097	-0.04	0.01	-2.91	.004
NDVI	-0.06	0.55	-0.10	.917	0.14	0.36	0.39	.695	-0.37	0.73	-0.51	.609	0.99	0.98	1.01	.313	0.31	0.70	0.44	.657
ISS	<0.01	0.04	0.09	.930	<0.01	0.03	0.15	.884	0.01	0.05	0.24	.806	0.12	0.06	2.12	.034	0.10	0.05	2.08	.038
Age	0.01	0.01	2.22	.026	0.01	0.00	3.38	.001	0.01	0.01	1.15	.249	-0.01	0.01	-0.55	.582	-0.02	0.01	-1.98	.048
Income	-0.21	0.06	-3.28	.001	-0.11	0.04	-2.86	.004	0.04	0.07	0.60	.548	-0.27	0.12	-2.28	.023	-0.09	0.07	-1.15	.251
ADI	<0.01	0.00	1.31	.190	<0.01	0.00	1.49	.136	<0.01	0.00	0.18	.855	0.01	0.01	1.95	.052	0.00	0.00	-0.09	.928
Marital Status [*unmarried*]	-0.27	0.21	-1.25	.213	<0.01	0.13	-0.01	.998	-0.17	0.27	-0.63	.528	<0.01	0.36	0.01	.993	-0.29	0.29	-1.02	.307
Childhood maltreatment	0.07	0.01	9.61	<.001	0.05	0.01	9.23	<.001	0.05	0.01	4.60	<.001	0.06	0.01	5.30	<.001	0.05	0.01	4.75	<.001
Head injury [*did not hit head*]	0.43	0.15	2.78	.005	0.33	0.10	3.33	.001	0.08	0.21	0.40	.686	0.45	0.27	1.68	.093	0.18	0.19	0.94	.348
LEC-5	0.05	0.01	6.19	<.001	0.03	0.01	6.08	<.001	0.01	0.01	1.03	.301	0.03	0.01	2.12	.035	0.01	0.01	0.87	.385

Abbreviations: **ADI:** Area Deprivation Index (national ranking); **CD-RISC:** Connor-Davidson Resilience Scale (total score); **ISS***:* Injury Severity Score; **LEC-5:** Life Events Checklist for DSM-5 (total score); **NDVI:** Normalized Difference Vegetation Index. *Notes:* continuous measures were grand-mean centered in the full sample; the reference group for dichotomous variables is provided in brackets; **bolded** numbers correspond to uncorrected $p < .05$.

© 2024 Webb EK et al. *JAMA Psychiatry.*

eTable9. Self-report and geocoded variables associated with class membership (full sample; interaction term; adjusting for baseline PTSD and medication use)

Variable	Trajectory Class *(statistical tests relative to the resilient trajectory)*																			
	**Nonremitting high**				**Nonremitting moderate**				**Delayed**				**Slow recovery**				**Rapid recovery**			
	Coefficient	*SE*	*Wald Z*	*p-value*	*Coefficient*	*SE*	*Wald Z*	*p-value*	*Coefficient*	*SE*	*Wald Z*	*p-value*	*Coefficient*	*SE*	*Wald Z*	*p-value*	*Coefficient*	*SE*	*Wald Z*	*p-value*
Intercept	-2.39	0.18	-13.08	<.001	-1.00	0.11	-8.94	<.001	-2.32	0.20	-11.37	<.001	-3.58	0.31	-11.69	<.001	-2.52	0.21	-11.93	<.001
Sex at Birth [*male*]	0.36	0.17	2.11	.035	0.55	0.11	5.09	<.001	-0.07	0.21	-0.34	.730	0.33	0.28	1.18	.237	0.40	0.21	1.97	.048
CD-RISC	-0.08	0.01	-7.25	<.001	-0.04	0.01	-5.77	<.001	-0.03	0.01	-2.17	.030	-0.02	0.02	-1.41	.157	-0.03	0.01	-2.76	.006
NDVI	-0.54	0.62	-0.88	.381	0.20	0.38	0.53	.593	-0.31	0.74	-0.41	.679	1.01	1.01	1.00	.316	0.34	0.71	0.48	.628
ISS	0.00	0.04	-0.07	.946	0.00	0.03	-0.12	.901	0.01	0.05	0.14	.885	0.12	0.06	1.96	.050	0.09	0.05	1.90	.058
Age	0.01	0.01	1.47	.141	0.01	0.00	2.73	.006	0.01	0.01	0.95	.343	-0.01	0.01	-0.80	.424	-0.02	0.01	-2.05	.041
Income	-0.07	0.07	-1.00	.317	-0.04	0.04	-1.01	.312	0.08	0.07	1.12	.263	-0.15	0.12	-1.28	.201	-0.04	0.08	-0.51	.613
ADI	0.00	0.00	0.72	.474	0.00	0.00	0.97	.333	0.00	0.00	0.12	.905	0.01	0.01	1.76	.078	0.00	0.00	-0.30	.767
Marital Status [*unmarried*]	-0.33	0.23	-1.44	.151	-0.01	0.14	-0.04	.970	-0.18	0.27	-0.64	.523	-0.07	0.37	-0.19	.851	-0.29	0.29	-1.00	.317
Childhood maltreatment	0.04	0.01	5.11	<.001	0.03	0.01	5.81	<.001	0.04	0.01	3.49	<.001	0.04	0.01	3.13	.002	0.04	0.01	3.50	<.001
Head injury [*did not hit head*]	0.19	0.16	1.17	.242	0.22	0.10	2.15	.031	0.02	0.21	0.07	.941	0.27	0.27	0.97	.330	0.12	0.20	0.62	.533
LEC-5	0.04	0.01	5.58	<.001	0.03	0.01	5.82	<.001	0.01	0.01	1.00	.319	0.03	0.01	2.06	.040	0.01	0.01	0.88	.376
Pre PCL-5 symptoms	0.08	0.01	14.61	<.001	0.05	0.00	12.02	<.001	0.03	0.01	3.89	<.001	0.07	0.01	7.61	<.001	0.03	0.01	4.71	<.001
Medication use [*no medications*]	0.01	0.20	0.04	.968	0.10	0.14	0.75	.450	0.19	0.27	0.69	.488	0.24	0.33	0.72	.471	-0.06	0.27	-0.20	.838
NDVI x CD-RISC	-0.24	0.08	-3.09	.002	-0.09	0.05	-1.71	.087	-0.02	0.10	-0.21	.833	-0.25	0.13	-1.93	.053	-0.13	0.09	-1.40	.160

Abbreviations: **ADI:** Area Deprivation Index (national ranking); **CD-RISC:** Connor-Davidson Resilience Scale (total score); **ISS***:* Injury Severity Score; **LEC-5:** Life Events Checklist for DSM-5 (total score); **NDVI:** Normalized Difference Vegetation Index. *Notes:* continuous measures were grand-mean centered in the full sample; the reference group for dichotomous variables is provided in brackets; **bolded** numbers correspond to uncorrected $p < .05$.

© 2024 Webb EK et al. *JAMA Psychiatry.*

eTable10. Self-report and geocoded variables associated with class membership (full sample; with Income x CD-RISC interaction)

	**Trajectory Class** *(statistical tests relative to the resilient trajectory)*																			
	**Nonremitting High**				**Nonremitting Moderate**				**Delayed**				**Slow recovery**				**Rapid recovery**			
**Variable**	*Coefficient*	*SE*	*Wald Z*	*p-value*	*Coefficient*	*SE*	*Wald Z*	*p-value*	*Coefficient*	*SE*	*Wald Z*	*p-value*	*Coefficient*	*SE*	*Wald Z*	*p-value*	*Coefficient*	*SE*	*Wald Z*	*p-value*
Intercept	-2.32	0.17	-13.35	<.001	-1.05	0.11	-9.75	<.001	-2.37	0.20	-11.79	<.001	-3.59	0.30	-11.81	<.001	-2.59	0.21	-12.43	<.001
Sex at Birth [*male*]	0.33	0.16	2.06	.039	0.53	0.10	5.08	<.001	-0.08	0.21	-0.37	.709	0.32	0.27	1.16	.247	0.36	0.20	1.80	.072
CD-RISC	-0.09	0.01	-8.99	<.001	-0.05	0.01	-6.82	<.001	-0.03	0.01	-2.53	.011	-0.05	0.02	-2.75	.006	-0.04	0.01	-3.23	.001
Income	-0.30	0.07	-4.05	<.001	-0.09	0.04	-2.24	.025	0.07	0.07	0.89	.374	-0.31	0.13	-2.42	.015	-0.07	0.08	-0.94	.348
NDVI	0.02	0.55	0.03	.977	0.15	0.36	0.40	.686	-0.35	0.73	-0.47	.636	1.06	0.98	1.08	.280	0.33	0.70	0.47	.637
ISS	0.01	0.04	0.25	.802	0.01	0.03	0.32	.750	0.02	0.05	0.36	.721	0.13	0.06	2.24	.025	0.10	0.05	2.17	.030
Age	0.01	0.01	2.30	.021	0.01	0.00	3.45	.001	0.01	0.01	1.22	.222	0.00	0.01	-0.42	.673	-0.02	0.01	-1.91	.056
ADI	0.00	0.00	1.08	.280	0.00	0.00	1.43	.154	0.00	0.00	0.13	.895	0.01	0.01	1.84	.065	0.00	0.00	-0.15	.884
Marital Status [*unmarried*]	-0.27	0.22	-1.23	.217	0.00	0.13	0.03	.976	-0.17	0.27	-0.62	.534	0.01	0.36	0.02	.987	-0.29	0.29	-1.00	.315
Childhood maltreatment	0.07	0.01	9.71	<.001	0.05	0.01	9.30	<.001	0.05	0.01	4.69	<.001	0.07	0.01	5.39	<.001	0.05	0.01	4.83	<.001
Head injury [*did not hit head*]	0.44	0.16	2.81	.005	0.34	0.10	3.40	.001	0.09	0.21	0.45	.650	0.46	0.27	1.68	.092	0.19	0.20	0.98	.329
LEC-5	0.05	0.01	6.41	<.001	0.03	0.01	6.23	<.001	0.01	0.01	1.19	.234	0.03	0.01	2.30	.021	0.01	0.01	1.00	.316
Income x CD-RISC	-0.05	0.01	-5.73	<.001	-0.02	<.001	-4.58	<.001	-0.03	0.01	-3.06	.002	-0.05	0.01	-3.75	<.001	-0.03	0.01	-2.89	.004

Abbreviations: **ADI:** Area Deprivation Index (national ranking); **CD-RISC:** Connor-Davidson Resilience Scale (total score); **ISS***:* Injury Severity Score; **LEC-5:** Life Events Checklist for DSM-5 (total score); **NDVI:** Normalized Difference Vegetation Index. *Notes:* continuous measures were grand-mean centered in the full sample; the reference group for dichotomous variables is provided in brackets; **bolded** numbers correspond to uncorrected $p < .05$.

© 2024 Webb EK et al. *JAMA Psychiatry.*

eTable11. Self-report and geocoded variables associated with class membership (MRI sample; with NDVI x CD-RISC interaction)

	**Trajectory Class** *(statistical tests relative to the resilient trajectory)*															
	**Nonremitting High**				**Nonremitting Moderate**				**Delayed**				**Recovery***			
**Variable**	*Coefficient*	*SE*	*Wald Z*	*p-value*	*Coefficient*	*SE*	*Wald Z*	*p-value*	*Coefficient*	*SE*	*Wald Z*	*p-value*	*Coefficient*	*SE*	*Wald Z*	*p-value*
Intercept	-5.14	1.10	-4.67	<.001	-1.49	0.37	-4.00	<.001	-2.73	0.61	-4.44	<.001	-3.48	0.74	-4.69	<.001
Sex at Birth [*male*]	1.88	0.86	2.17	.030	0.62	0.35	1.79	.074	0.15	0.54	0.28	.781	0.48	0.60	0.80	.425
CD-RISC	-0.20	0.05	-4.16	<.001	-0.10	0.03	-3.61	<.001	-0.08	0.04	-2.02	.044	-0.07	0.04	-1.72	.086
NDVI	1.90	3.10	0.61	.539	-1.60	1.18	-1.36	.174	-0.29	2.00	-0.15	.883	2.95	2.11	1.40	.162
ISS	-0.12	0.21	-0.56	.575	0.04	0.09	0.40	.689	-0.09	0.16	-0.53	.598	0.03	0.16	0.21	.834
Age	0.03	0.02	1.09	.276	0.00	0.01	0.04	.967	0.01	0.02	0.51	.610	-0.05	0.03	-1.69	.090
Income	-0.05	0.25	-0.19	.847	-0.06	0.12	-0.51	.612	-0.01	0.18	-0.05	.960	-0.05	0.19	-0.26	.792
ADI	0.00	0.01	-0.04	.969	0.01	0.01	2.15	.032	0.01	0.01	0.66	.511	0.00	0.01	-0.15	.883
Marital Status [*unmarried*]	-14.25	0.00	-2.06E+06	<.001	0.08	0.50	0.15	.878	0.42	0.76	0.55	.582	0.74	0.87	0.86	.392
Childhood maltreatment	0.05	0.03	1.99	.047	0.04	0.02	2.29	.022	-0.03	0.03	-0.76	.446	0.05	0.03	2.13	.033
Head injury [*did not hit head*]	1.40	0.70	2.01	.045	0.28	0.32	0.88	.380	0.48	0.54	0.89	.375	0.84	0.61	1.39	.166
LEC-5	0.07	0.03	2.07	.038	0.04	0.02	1.99	.046	0.00	0.03	0.07	.941	0.03	0.03	1.10	.272
NDVI x CD-RISC	0.02	0.33	0.06	.953	-0.22	0.16	-1.36	.173	0.07	0.26	0.29	.775	0.23	0.25	0.91	.365

Abbreviations: **ADI:** Area Deprivation Index (national ranking); **CD-RISC:** Connor-Davidson Resilience Scale (total score); **ISS***:* Injury Severity Score; **LEC-5:** Life Events Checklist for DSM-5 (total score); **NDVI:** Normalized Difference Vegetation Index. *Notes:* continuous measures were grand-mean centered in the full sample; the reference group for dichotomous variables is provided in brackets; **bolded** numbers correspond to uncorrected $p < .05$; * rapid and slow recovery groups from the full trajectory analysis were combined.

© 2024 Webb EK et al. *JAMA Psychiatry.*

eTable12. General linear models for reward reactivity

Region	*Standardized Coefficient*	*t-statistic*	*Uncorrected p-value*
**Amygdala**			
Intercept	-	3.86	<.001
Sex at Birth [*male*]	-0.02	-0.33	0.745
CD-RISC	0.00	-0.01	0.994
**NDVI**	**0.18**	**2.83**	**0.005**+
ISS	-0.01	-0.20	0.840
Age	-0.11	-1.71	0.088
Income	0.02	0.36	0.717
ADI	0.07	1.06	0.290
Marital Status [*unmarried*]	0.06	0.93	0.351
Childhood maltreatment	-0.02	-0.27	0.784
LEC-5	-0.04	-0.67	0.506
**Nucleus Accumbens**			
Intercept	-	8.52	0.000
Sex at Birth [*male*]	0.01	0.13	0.899
CD-RISC	0.11	1.78	0.077
NDVI	0.11	1.71	0.088
ISS	-0.06	-1.02	0.309
Age	-0.02	-0.27	0.791
Income	0.05	0.77	0.441
ADI	0.10	1.62	0.107
Marital Status [*unmarried*]	-0.02	-0.22	0.825
Childhood maltreatment	0.06	0.97	0.332
LEC-5	0.04	0.67	0.502
**Orbitofrontal Cortex**			
Intercept	-	1.59	0.113
Sex at Birth [*male*]	-0.08	-1.40	0.163
CD-RISC	0.01	0.15	0.881
NDVI	0.05	0.76	0.450
ISS	-0.01	-0.09	0.930
Age	0.07	1.10	0.272
Income	0.08	1.15	0.250
ADI	0.06	0.94	0.351
Marital Status [*unmarried*]	0.05	0.76	0.450
Childhood maltreatment	0.10	1.49	0.136
LEC-5	-0.09	-1.50	0.136

Abbreviations: **ADI:** Area Deprivation Index (national ranking); **CD-RISC:** Connor-Davidson Resilience Scale (total score); **ISS***:* Injury Severity Score; **LEC-5:** Life Events Checklist for DSM-5 (total score); **NDVI:** Normalized Difference Vegetation Index. *Notes:* continuous measures were grand-mean centered in the full sample; the reference group for dichotomous variables is provided in brackets; **bolded** numbers correspond to uncorrected $p < .05$.+ Survived correction for multiple comparisons.

© 2024 Webb EK et al. *JAMA Psychiatry.*

eTable13. General linear models for reward reactivity

Region	*Standardized Coefficient*	*t-statistic*	*Uncorrected p-value*
**Amygdala**			
Intercept	-	3.92	<.001
Sex at Birth [*male*]	-0.02	-0.38	0.705
CD-RISC	-0.02	-0.26	0.794
**NDVI**	**0.18**	**2.88**	**0.004**
ISS	-0.01	-0.24	0.808
Age	-0.11	-1.64	0.102
Income	0.03	0.39	0.701
ADI	0.07	1.09	0.279
Marital Status [*unmarried*]	0.07	0.95	0.343
Childhood maltreatment	-0.02	-0.27	0.788
LEC-5	-0.04	-0.73	0.467
NDVI x CDRISC	-0.07	-1.10	0.273
**Nucleus Accumbens**			
Intercept	-	8.46	<.001
Sex at Birth [*male*]	0.01	0.15	0.880
CD-RISC	0.12	1.84	0.067
NDVI	0.11	1.69	0.093
ISS	-0.06	-1.00	0.319
Age	-0.02	-0.30	0.768
Income	0.05	0.76	0.447
ADI	0.10	1.60	0.110
Marital Status [*unmarried*]	-0.02	-0.23	0.820
Childhood maltreatment	0.06	0.97	0.334
LEC-5	0.04	0.70	0.485
NDVI x CDRISC	0.03	0.50	0.618
**Orbitofrontal Cortex**			
Intercept	-	1.60	0.111
Sex at Birth [*male*]	-0.08	-1.41	0.16
CD-RISC	0.01	0.08	0.934
NDVI	0.05	0.77	0.444
ISS	-0.01	-0.10	0.922
Age	0.07	1.11	0.267
Income	0.08	1.16	0.249
ADI	0.06	0.94	0.349
Marital Status [*unmarried*]	0.05	0.76	0.449
Childhood maltreatment	0.10	1.49	0.137
LEC-5	-0.09	-1.51	0.133
NDVI x CDRISC	-0.02	-0.27	0.788

Abbreviations: **ADI:** Area Deprivation Index (national ranking); **CD-RISC:** Connor-Davidson Resilience Scale (total score); **ISS***:* Injury Severity Score; **LEC-5:** Life Events Checklist for DSM-5 (total score); **NDVI:** Normalized Difference Vegetation Index. *Notes:* continuous measures were grand-mean centered in the full sample; the reference group for dichotomous variables is provided in brackets; uncorrected ***** $p < .05$.

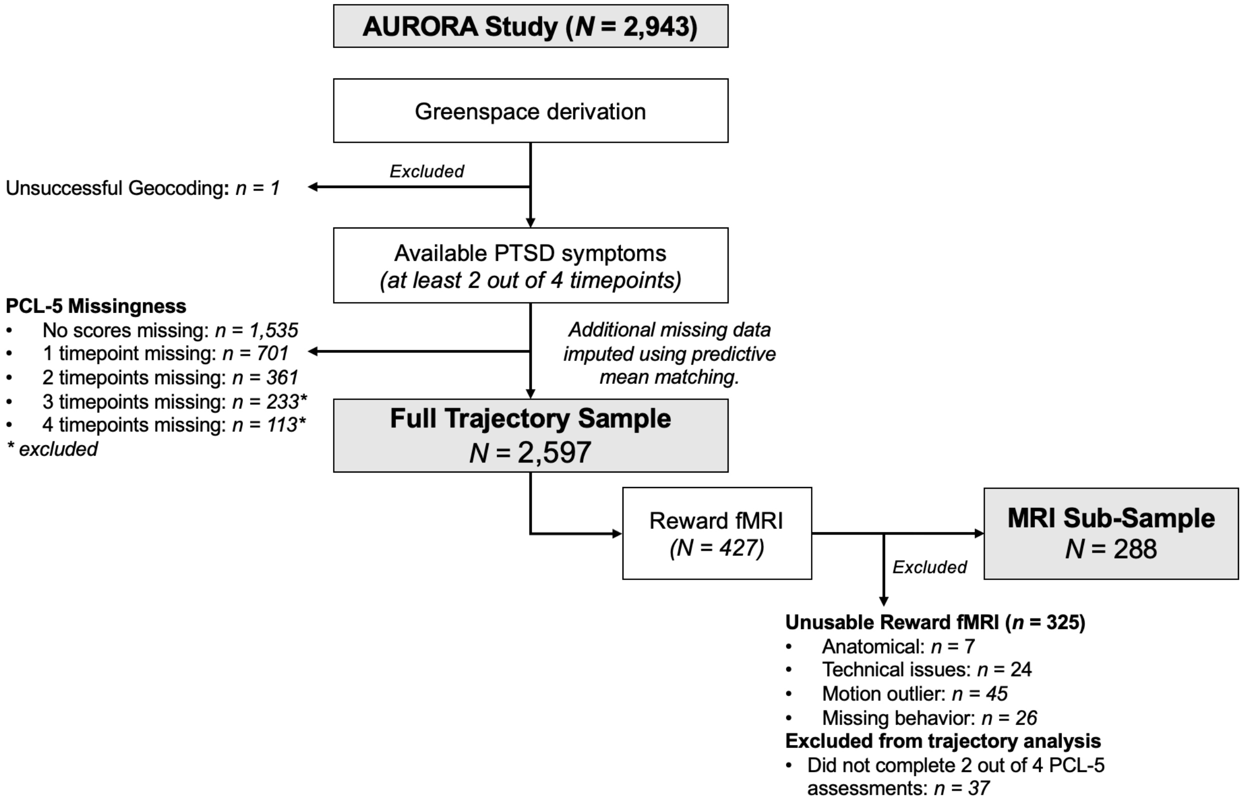

eFigure1. Flowchart of AURORA study participants who met inclusion criteria for the full trajectory analysis or the fMRI reward reactivity analyses.

© 2024 Webb EK et al. *JAMA Psychiatry.*

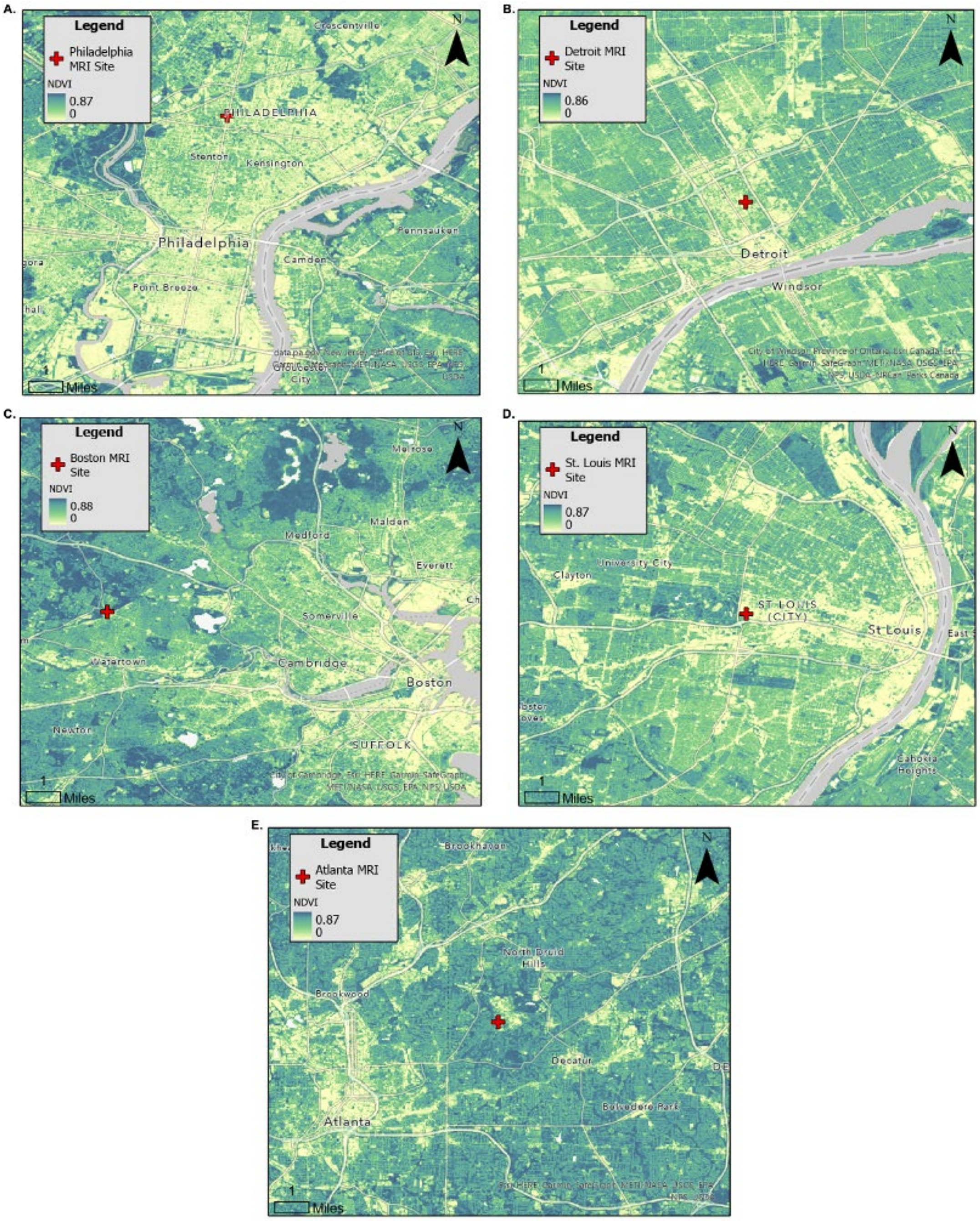

eFigure2. Greenspace (between May 1st, 2017, through September 30th, 2017), as quantified by the Normalized Difference Vegetation Index (NDVI), surrounding each of the five study scan sites near **[A]** Philadelphia, Pennsylvania, **[B]** Detroit, Michigan, **[C]** Boston, Massachusetts, **[D]** St. Louis, Missouri, and **[E]** Atlanta, Georgia.

© 2024 Webb EK et al. *JAMA Psychiatry.*

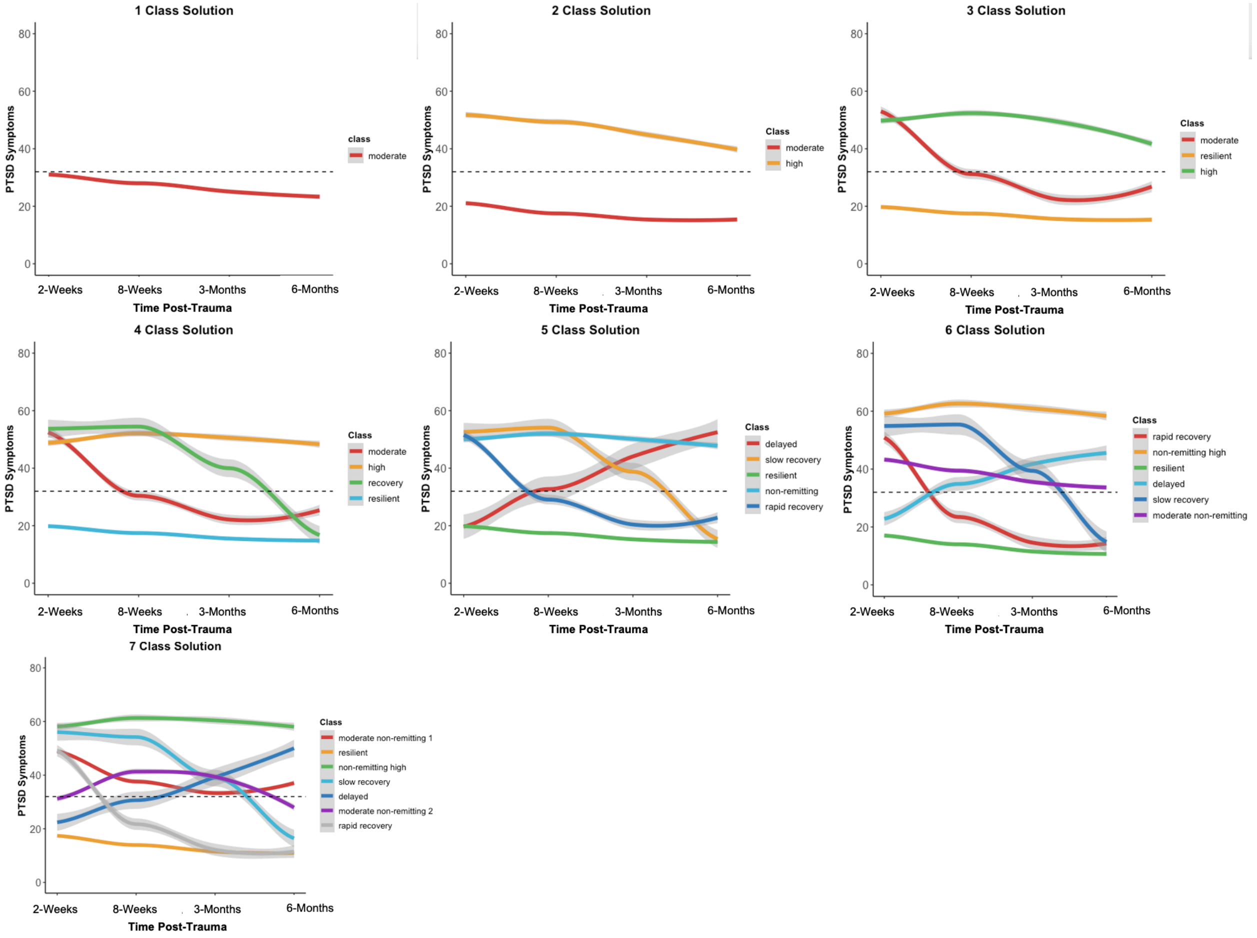

eFigure3. Results of the latent class mixed effect models with 1 to 7 classes. The 6-class solution fit well (see fit indices in Table 2 of main text), was parsimonious, and had strong theoretical justification.

© 2024 Webb EK et al. *JAMA Psychiatry.*

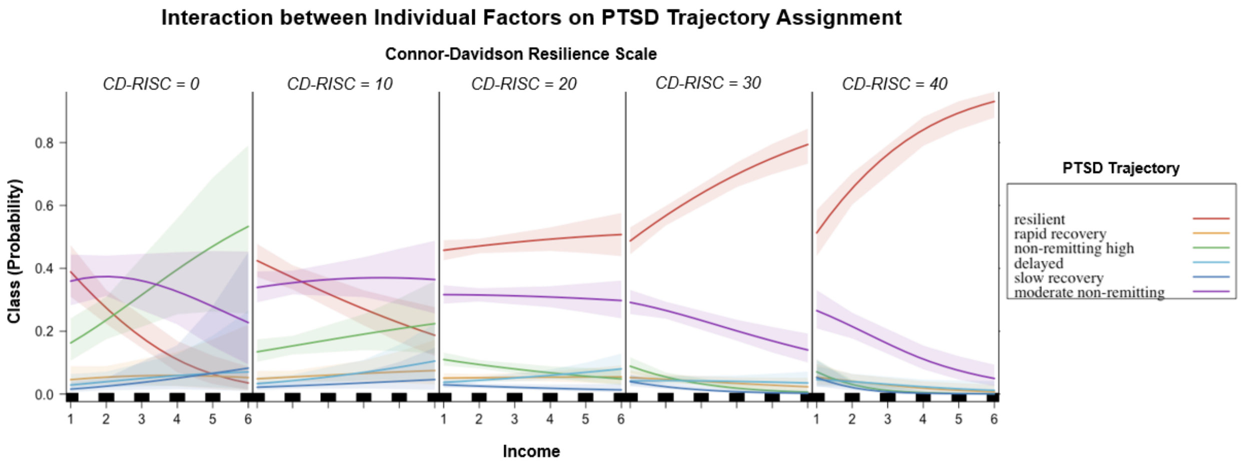

eFigure4. There was a significant interaction between income and CD-RISC scores in predicting class, such that individuals reporting higher levels of perceived internal resources with higher income had an even greater likelihood of assignment in the resilient trajectory compared to the nonremitting high, nonremitting moderate, delayed, rapid recovery, and slow recovery classes.

© 2024 Webb EK et al. *JAMA Psychiatry.*
