# Supplementary material for: Neighborhood Resources Associated With Psychological Trajectories and Neural Reactivity to Reward After Trauma
Source: JAMA Psychiatry. 2024 Jul 31;81(11):1090–100. doi: 10.1001/jamapsychiatry.2024.2148 (PMC11292566; doi:10.1001/jamapsychiatry.2024.2148)
Supplement: Supplement 2. — Data Sharing Statement. [file jamapsychiatry-e242148-s002.pdf]

# Data Sharing Statement

Webb. Neighborhood Resources Associated With Psychological Trajectories and Neural Reactivity to Reward After Trauma. *JAMA Psychiatry*. Published July 31, 2024. doi:10.1001/jamapsychiatry.2024.2148

**Data**
**Data available:** Yes
**Data types:** Deidentified participant data
**How to access data:** Data and/or research tools used in the preparation of this manuscript were obtained from the National Institute of Mental Health (NIMH) Data Archive (NDA). NDA is a collaborative informatics system created by the National Institutes of Health to provide a national resource to support and accelerate research in mental health. Dataset identifier(s): NIMH Data Archive Digital Object Identifier (DOI) 10.15154/wyh4-xr50. This manuscript reflects the views of the authors and may not reflect the opinions or views of the NIH or of the Submitters submitting original data to NDA.
**When available:** With publication

**Supporting Documents**
**Document types:** None

**Additional Information**
**Who can access the data:** Individuals can access this data without researchers approval through through National Institute of Mental Health (NIMH) Data Archive (NDA). Dataset identifier(s): NIMH Data Archive Digital Object Identifier (DOI) 10.15154/wyh4-xr50.
**Types of analyses:** Individuals can access this data without researchers approval through through National Institute of Mental Health (NIMH) Data Archive (NDA). Dataset identifier(s): NIMH Data Archive Digital Object Identifier (DOI) 10.15154/wyh4-xr50.
**Mechanisms of data availability:** Individuals can access this data without researchers approval through through National Institute of Mental Health (NIMH) Data Archive (NDA). Dataset identifier(s): NIMH Data Archive Digital Object Identifier (DOI) 10.15154/wyh4-xr50.
